# Study of Photoselectivity in Linear Conjugated Chromophores Using the XMS-CASPT2 Method

**DOI:** 10.1021/acsphyschemau.4c00065

**Published:** 2024-10-02

**Authors:** Saumik Sen, Xavier Deupi

**Affiliations:** † Condensed Matter Theory Group, Laboratory for Theoretical and Computational Physics, Center for Scientific Computing, Theory, and Data, Paul Scherrer Institute, 5232 Villigen, Switzerland; ‡ Laboratory of Biomolecular Research, Center for Life Sciences, Paul Scherrer Institute, 5232 Villigen, Switzerland; § Swiss Institute of Bioinformatics (SIB), 1015 Lausanne, Switzerland

**Keywords:** photoisomerization, photoselectivity, retinal chromophore, photoswitches, conical intersection, XMS-CASPT2

## Abstract

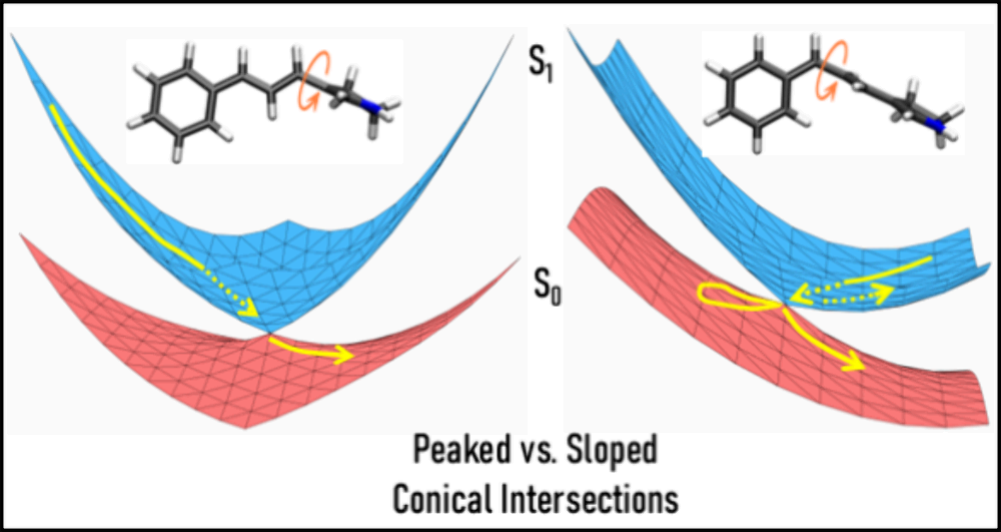

Photoisomerization, the structural alteration of molecules upon absorption of light, is crucial for the function of biological chromophores such as retinal in opsins, proteins vital for vision and other light-sensitive processes. The intrinsic selectivity of this isomerization process (i.e., which double bond in the chromophore is isomerized) is governed by both the inherent properties of the chromophore and its surrounding environment. In this study, we employ the extended multistate complete active space second-order perturbation theory (XMS-CASPT2) method to investigate photoisomerization selectivity in linear conjugated chromophores, focusing on two simple molecular models resembling retinal. By analyzing electronic energies, intramolecular charge separation, and conical intersection topographies in the gas phase, we show that the photoproduct formed by rotation around the double bond near the Schiff base is energetically favored. The topographic differences at the conical intersections leading to different photoproducts reveal differences in photodynamics. In multiphoton excitation, the primary photoproduct typically reverts to the initial configuration rather than rotating around a different double bond. Our study offers new insights into the photodynamics of photoisomerizing double bonds in π-conjugated chromophores. We anticipate that our findings will provide valuable perspectives for advancing the understanding of biological chromophores and for designing efficient photochemical switches with applications in molecular electronics and phototherapy.

## 1 Introduction

**Figure 1:**
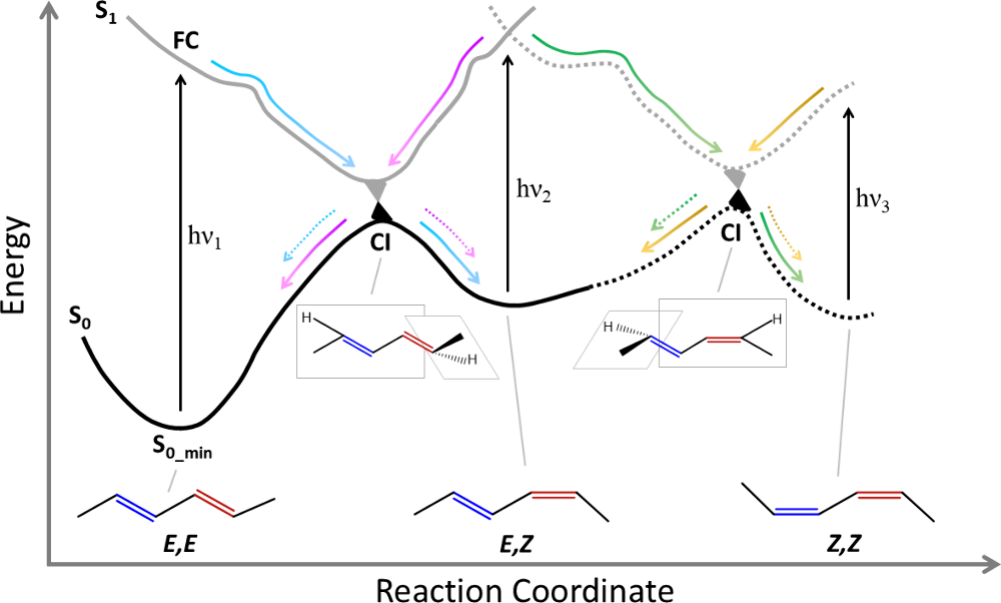
Schematic representation of sequential and reversible photoisomerization processes in 2,4-hexadiene upon absorption of multiple photons (*h*ν_1_, *h*ν_2_, *h*ν_3_). S_0_ and S_1_ depict the energies of the ground and the first electronically excited states along the isomerization reaction coordinate. A first photon *h*ν_1_ can isomerize the initial (*E*,*E*) form to the photoproduct (*E*,*Z*) (cyan path). A second photon *h*ν_2_ can lead to the formation of the photoproduct (*Z*,*Z*) (green path) or, alternatively, revert the first isomerization to yield the original (*E*,*E*) form (magenta path). A third photon *h*ν_3_ absorbed by (*Z*,*Z*) could similarly lead to the (*E*,*Z*) form (yellow path).

Photoisomerization is a chemical reaction in which a molecule undergoes a structural change upon absorption of a photon. The absorption of multiple photons can lead to sequential or reversible photoisomerization reactions. For instance, the linear conjugated 2,4-hexadiene could successively isomerize between (*E*,*E*), (*E*,*Z*), and (*Z*,*Z*) conformations (Figure 1).

**Figure 2:**
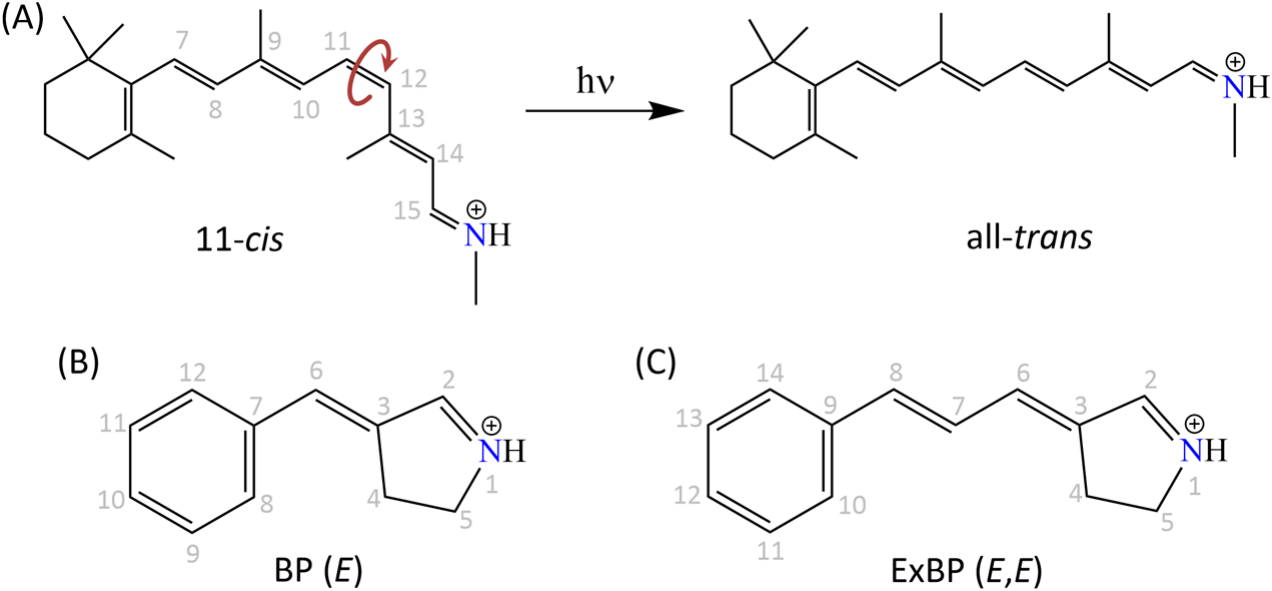
(A) Isomerization of the retinal protonated Schiff base from the 11-*cis* to the all-*trans* isomer upon photoexcitation. (B) Structure of the *E* isomer of 4-benzylidene-3,4-dihydro-2*H*-pyrrolium (BP). (C) Structure of the (*E*,*E*) isomer of the extended conjugated (by one double bond) model of BP (ExBP).

Photoisomerization reactions are highly valuable to many industrial and medical applications, such as molecular electronics, information storage, and drug delivery.^1−4^ In biology, one of the classic examples of molecular photoswitches is the retinal protonated Schiff base (PSB) chromophore (Figure 2A) of rhodopsin, which initiates the cellular signal transduction processes that ultimately lead to vision.^5−8^ The extended conjugation of retinal and related photoswitches allows the absorption of light over a wide range of wavelengths. An extended conjugated polyene also allows photoisomerization of different double bonds. The selectivity of isomerization (i.e., which double bond is isomerized) is a critical factor that determines the outcome of the reaction. For instance, photoisomerization of the retinal PSB in rhodopsins is generally specific to certain double bonds. In animal rhodopsins, retinal typically isomerizes from 11-*cis* to all-*trans* (Figure 2A), although in some invertebrate opsins – such as in jumping spider rhodopsin 1 – it can isomerize from the 9-*cis* isomer.^9,10^ In microbial rhodopsins, the retinal PSB usually undergoes all-*trans* to 13-*cis* isomerization.^11−14^ Recently, all-*trans* to 11-*cis* isomerization has been reported in the microbial bestrhodopsin,^15^ and an extremely rare photoisomerization of all-*trans* to 7-*cis* has been observed in a near-infrared light-absorbing enzymerhodopsin.^16^

In solution, photoisomerization of retinal is not strongly selective and can occur at various double bonds with low quantum yields and reduced reaction rates.^17−21^ Thus, the selectivity of isomerization seems to arise in the protein binding pocket, where it could be controlled by various factors. For instance, the electronic properties of nearby amino acids influence the electron density along the conjugated system of retinal. As a result, some double bonds are more electron-rich (or electron-poor) than others, leading to a certain variance in bond lengths and orders. This affects the energy required to break the double bond and initiate the isomerization reaction. In addition, inherent asymmetries in the structure of the chromophore, assisted by van-der-Waals interactions with nearby amino acids in a tightly constrained binding pocket, contribute to regulating isomerization selectivity and also the direction of rotation.

The selectivity in retinal PSB photoisomerization has been investigated in the past using theoretical/computational methods. For instance, a hybrid quantum mechanics/molecular mechanics (QM/MM) study (using CASPT2//CASSCF/6-31G*/AMBER) of the excited-state relaxation paths of retinal in isorhodopsin (9-*cis* to all-*trans*) and rhodopsin (11-*cis* to all-*trans*) has shown that both isomers relax along a common excited-state potential energy valley, although the energetics marginally favor isomerization from 11-*cis*.^22^ Another QM/MM study (using the B3LYP/6-31G* for the retinal PSB and AMBER for the protein) calculated energies and absorption wavelengths for different possible photoisomerizations.^23^ Despite these (and other) works, QM/MM studies of the retinal chromophore inside the protein remain very challenging due to the high number of reaction coordinates associated with the photoisomerization pathway. Moreover, theoretical studies of retinal photoisomerization pose a particular challenge to traditional quantum chemical methods, as this process involves a strong coupling between nuclear and electronic motion. This results in a breakdown of the Born–Oppenheimer approximation, especially near the conical intersection (CI) point where electronic energies degenerate and exhibit a strong multireference character. Thus, more advanced ab initio QM studies have been performed in the gas phase – i.e., without considering the protein environment – and using simplified models of the retinal PSB. Nonadiabatic dynamics using CASSCF(6,6)/6-31G on a model 9-*cis* retinal PSB showed that an energetic barrier in the excited state impedes the *cis*-*trans* isomerization.^24,25^ Recent studies on an isolated retinal PSB, employing the XMS-CASPT2 and MRPT2 methods, have concluded that the minimum energy conical intersection (MECI) leading to 13=14 bond isomerization is energetically inaccessible in comparison to the 11=12 bond.^26−28^ Moreover, CASPT2//CASSCF studies on an isolated retinal PSB suggested that bond selectivity is not solely determined by energetic considerations in the absence of specific environmental interactions,^29^ but also by the topographies (peaked vs sloped) of the ground and excited state potential energy surfaces near the conical intersection. Specifically, the authors found that the CI for the isomerization of the 13=14 bond has a sloped topography while for the 11=12 bond, it is strongly peaked. A peaked CI is more effective in steering the excited state population away from the intersection to the ground state. Conversely, in a sloped intersection, the excited state population is less directed, leading to a higher probability of up-funneling from S_0_ to S_1_.^29−31^ Very recently, these differences in topography of the conical intersection have been used to explain the different orders of excited state lifetimes for two biomimetic photothermal molecules.^32^ These studies clearly indicate that, in addition to the effects of the surrounding amino acids in the protein binding pocket, there are intrinsic factors within the chromophore – not yet fully understood – that also regulate the selectivity of isomerization. In a protein environment, the surroundings likely exert a more significant influence; however, the inherent traits of the chromophore should persist during the reaction, possibly priming a preferred isomerization. Such traits are revealed more prominently in calculations performed in the gas phase, where external influences are minimized, allowing a clearer assessment of the inherent properties governing isomerization selectivity.

Understanding the energetics and structural dynamics associated with *E*/*Z* photoisomerization in linear conjugated systems is crucial to unravel its underlying mechanism and, thus, to facilitate the design of efficient photochemical reactions. For instance, the efficiency of the naturally occurring retinal chromophore in undergoing *E*/*Z* isomerization has been exploited to design novel photoswitches. These are particularly important due to their biological relevance, rapid light response, tunable properties, and reversible photocontrol which enables them to be applied in tailored applications.^33−36^ In this study, we have investigated the energetics of the selective rotation of the double bonds involved in photoisomerization and the associated structural changes that lead to the MECI in the model systems 4-benzylidene-3,4-dihydro-2*H*-pyrrolium (BP)^33^ (Figure 2B) and its extended conjugated model (ExBP) (Figure 2C). BP is a benzylidene-pyrroline chromophore resembling the retinal PSB but with the Schiff base locked within the pyrrolium ring, and has only one isomerizable double bond between two cyclic units. BP has been used to control light-switchable properties in peptides and proteins.^35,37^ For this study, we have created ExBP by inserting an additional conjugation in the polyene chain of BP. Thus, ExBP has two photoisomerizing central double bonds, which allows us to investigate isomerization selectivity in highly conjugated chromophores. Analysis of the redistribution of molecular charge and the topography near the conical intersection of BP and ExBP provide a better insight into the fundamental factors that regulate isomerization selectivity and photoproduct formation. We have used the extended multistate complete active space second-order perturbation theory (XMS-CASPT2), a multireference perturbation theory designed to accurately treat electronic correlation effects in excited states.^38,39^ The wave function generated from the complete active space self-consistent field (CASSCF) method is used as a reference wave function for the XMS-CASPT2 method.^40^ The implementation of analytic nuclear gradients and nonadiabatic coupling vectors in the XMS-CASPT2 method allows for the efficient optimization of molecular geometries and the coupling between different electronic states as the nuclei move, respectively.^41^ These are essential for exploring potential energy surfaces of the nonadiabatic photoisomerization processes. The XMS-CASPT2 method has been effectively used in accurately describing both the ground and electronically excited states, as well as conical intersections, of highly conjugated organic molecules and retinal analogs.^42−45^

## 2 Methodology

The XMS-CASPT2 method provides a powerful and accurate computational approach to studying excited states and nonadiabatic processes, making it highly valuable for exploring complex photoisomerization paths. It is an improvement over the original CASPT2 method,^46^ especially near the potential energy surface crossing regions (for example, at the conical intersection) as it is invariant with respect to unitary rotations of the reference functions.

The geometries of all the molecules considered in this work were optimized at the XMS-CASPT2 level of theory using the cc-pVDZ basis set along with the cc-pVDZ-JKFIT density fitting basis. The reference wave function was obtained from three state-averaged (SA3) CASSCF method with ten electrons in ten active π-orbitals (10o,10e) for BP, and 12 electrons in 12 active π-orbitals (12o,12e) for the extended model (ExBP) (Figure S10). The vertical excitation energies of three excited states were calculated on the XMS-CASPT2 geometries at XMS-CASPT2, CC2, and ADC(2) levels of theories.^47^ Both cc-pVDZ and cc-pVTZ basis sets were used for the excitation energy calculations to oversee the effect of a larger basis.^48^ In the case of BP, we have also optimized the geometries of both the *E* and *Z* isomers at the optically bright first excited state, and subsequently, the emission energies were evaluated.

The MECI along the photoisomerization path between the first excited state (S_1_) and the ground state (S_0_) was calculated using three state-averaged XMS-CASPT2/cc-pVDZ methodology. Both the analytic gradients and nonadiabatic coupling (NAC) vectors of the relevant electronic states are required to calculate the MECI. In the case of BP, the MECI was obtained using the gradient projection method by Bearpark, Robb and Schlegel by evaluating analytical gradients and NACs.^49^ In this method, the molecular gradient is expressed by the sum of the energy difference gradient and projection of the higher state gradient onto the *n* – 2 intersection space (*n* = number of degrees of freedom of the molecule) consisting of an infinite number of conical intersection points.^50^ However, for the extended model ExBP, the calculation of the MECI using both analytic gradients and NAC vectors was computationally too expensive; therefore, in this case the conical intersection was optimized using the CIOpt program without evaluating the NACs.^51^ A linear interpolation of internal coordinates (LIIC) between the Franck–Condon (FC) geometry and MECI point was achieved using the Columbus program,^52−54^ and subsequently, the energies for various geometries were calculated using the XMS-CASPT2/cc-pVDZ methodology. The Mulliken atomic charges were calculated from the one-particle relaxed density matrices.

2D potential energy surfaces were generated to explore the topography near the MECI by utilizing two orthonormalized branching plane vectors, namely the gradient difference vector (GDV; **g**) and the nonadiabatic coupling vector (NACV; **h**) between S_0_ and S_1_. The geometries were generated by displacing the MECI in steps of 0.01 Å up to 0.05 Å in each direction along the two orthonormalized branching plane vectors. Moreover, the MECI can be characterized by calculating the electronic state energies moving along the circular loop centered around the conical intersection point.^55^ Along the circular loop, if the conical intersection point is a minimum in the higher energy surface, then it is called a peaked intersection. However, if the energy of the higher state becomes lower than the conical intersection point, then it is a sloped intersection. Furthermore, the number of minima on the S_0_ energy surface represents preferred paths for the relaxation on the lower surface. A bifurcating intersection has two minima, while a single-path intersection has only one. Relaxation along other directions would be diverted toward one of these lower energy paths. The S_1_ energy surface also has the same number of minima as in S_0_, and they signify the preferred paths on the higher energy surface leading to the conical intersection point. We have calculated the energies of S_0_ and S_1_ in a circular loop constructed along the orthonormalized branching plane vectors around the minimum energy conical points, and the energies were plotted against the circular loop angles in an increment of 10 degrees for a radius of 0.001 Å. Additionally, the topology of the MECIs were classified by calculating the parameters described by Yarkony,^56,57^ and Fdez Galván et al.^55^

The single-state single reference (SS-SR) internal contraction scheme has been used for all the XMS-CASPT2 calculations.^58,59^ Moreover, a level shift parameter of 0.2 *E*_h_ was used to overcome the problem of intruder states.^60^ No imaginary shift was used in the calculations. All the XMS-CASPT2 calculations were performed using the BAGEL software package^61^ whereas RI-CC2 and RI-ADC(2) excitation energies were calculated with the Turbomole 7.5.1 program package.^62,63^

## 3 Results and Discussion

### 3.1 Investigation of BP

In BP, there is only one possible isomerization around the intercyclic double bond. Investigation of the photoisomerization profile of BP offers thus a simple model to analyze the factors associated with photoisomerization of more complex polyene systems, such as retinal chromophore in rhodopsins. The geometries of the *E* and *Z* isomers of BP were optimized using the XMS-CASPT2/cc-pVDZ methodology. The *E* isomer is planar while the *Z* isomer is about −7 and −48 degrees twisted at the central double (C2–C3=C6–C7) and single (C3=C6–C7=C8) bonds, respectively, due to the steric interaction of phenyl ring with the heterocyclic hydrogen atom. The twisted configuration of the *Z* isomer can induce chirality, which might result in unique optical and electronic properties. This leads to the destabilization of the *Z* isomer by 0.07 eV with respect to the *E* configuration. The excitation energies for *E* and *Z* isomers of BP are given in Table S1. For all the methods considered, the first excited state (S_1_) was found to be optically bright for both isomers and therefore photochemically accessible. However, the oscillator strength of the *E* isomer is much higher compared to the *Z* form. These structural and spectroscopic characteristics were also observed in UV/vis spectroscopy and quantum chemical calculations using CASPT2//CASSCF/6-31G*.^33^

**Figure 3:**
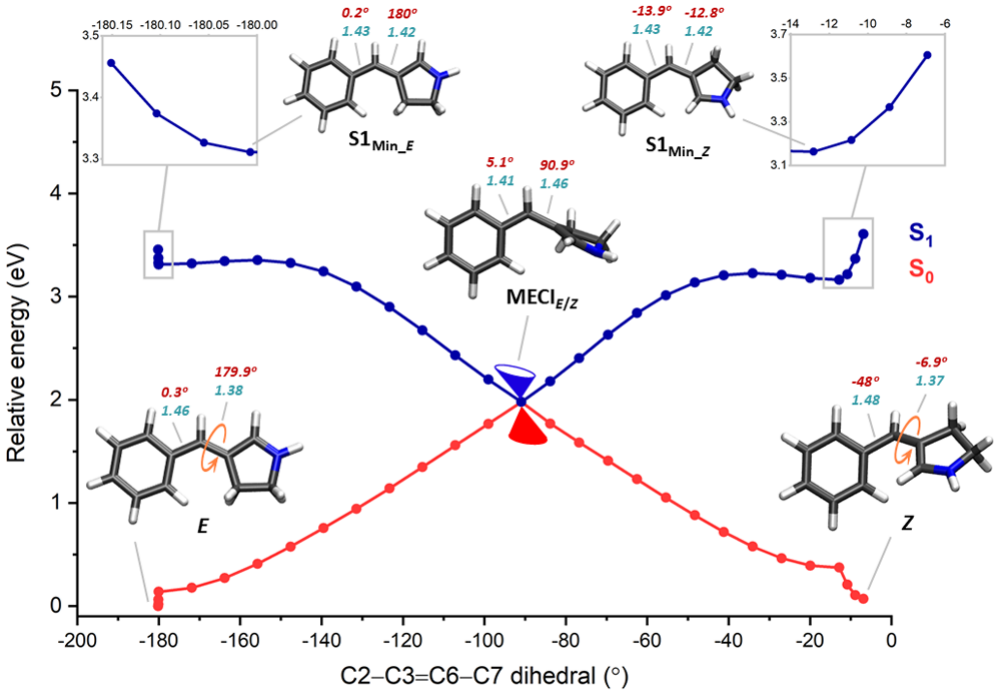
*E*/*Z* photoisomerization profile of BP and minimum energy structures. The dihedrals and bond lengths are shown in red and turquoise, respectively.

The minimum energy conical intersection (MECI_*E*/*Z*_) between the first excited state (S_1_) and the ground state (S_0_) was calculated at the XMS-CASPT2/cc-pVDZ level, which allowed us to generate the *E*/*Z* photoisomerization profile (Figure 3) passing through the MECI_*E*/*Z*_ point. Both *E* and *Z* isomers were also optimized at the optically bright first excited state (S_1_) to locate the minima in the S_1_ using the same level of theory. The emission energies were calculated and are provided in Table S2.

The LIIC method was used to connect the minimum energy geometries to the MECI_*E*/*Z*_ and subsequently, the energies were calculated using the XMS-CASPT2/cc-pVDZ level of theory. In the *E*-to-*Z* isomerization, the minimum energy configuration in the first excited state (S1_Min_*E*_) is stabilized by about 0.15 eV compared to the energy of the FC point (in the S_1_ state) of the *E* isomer. In the S1_Min_*E*_ the dihedral angles at the central double and single bonds are comparable to the *E* isomer, but the bond lengths get altered (the C3=C6 bond is elongated by 0.04 Å whereas the C6–C7 bond is contracted by 0.03 Å). In the context of retinal isomerization dynamics in rhodopsin, this phenomenon of bond length alternation (BLA), wherein bond lengths undergo inversion, constitutes the first contributor to the isomerization reaction coordinate. The S_1_ potential energy surface from S1_Min_*E*_ to the MECI passes through a small barrier of 0.04 eV. At the MECI_*E*/*Z*_, the central double and single bonds are twisted by about 91 and 5 degrees, respectively, signifying a double bond rotation via a one-bond-flip (OBF) mechanism.^64^ The dihedral twist represents the second reaction coordinate to the photoisomerization process. A pyramidalization of about 9 degrees is observed at the pyrrolium nitrogen in the MECI_*E*/*Z*_. Pyramidalization results in a sudden change in the polarizing effect at the MECI and thereby plays a crucial role in the photoisomerization dynamics of many conjugated chromophores by lowering the energy of the excited state.^31,44,65−67^ At MECI_*E*/*Z*_ the bond length of C3=C6 was elongated by 0.08 Å whereas the C6–C7 was contracted by 0.05 Å compared to the *E* isomer. In the case of *Z*-to-*E* isomerization, the profile connecting the *Z* isomer and the MECI_*E*/*Z*_ passes through a minimum at the first excited state (S1_Min_*Z*_) which is about −13 and −14 degrees twisted in the central double and single bonds and about 0.44 eV lower in energy compared to the S_1_ energy of the *Z* isomer in the FC point. In S1_Min_*Z*_, both the elongation of the C3=C6 bond length and the contraction of the C6–C7 bond occur by an equal measure of 0.05 Å in comparison to the *Z* isomer. In the *Z*-to-*E* isomerization, the S_1_ excited state path that connects S1_Min_*Z*_ to the MECI_*E*/*Z*_ has an energetic barrier of 0.06 eV. Therefore, in BP, the *E*-to-*Z* isomerization is photochemically more feasible than *Z*-to-*E*.

Photoisomerization in conjugated chromophores results in a redistribution of the molecular charge along the electronically conjugated chromophore via resonance. Therefore, we investigated the distribution of charge at each side of the central double bond (C3=C6 bond) of BP along the *E*/*Z* photoisomerization pathway. Figure S1A shows the distribution of ground and excited state charges for the *E* and *Z* isomers and the MECI_*E*/*Z*_ point. In the ground state, the positive charge is highly localized on the pyrrolium group mimicking the Schiff base (fragment 1). However, at the MECI_*E*/*Z*_ the charge is more evenly distributed. In the excited states, the electronic charge delocalizes and is slightly more concentrated near the benzylidene ring (fragment 2). The extent of charge separation in the S_2_ state is larger than in S_1_, especially at the MECI_*E*/*Z*_. Interestingly, the extent of charge transfer is higher in the photoexcitation of the *Z* isomer, which could possibly be related to its twisted conformation (twisted intramolecular charge transfer (TICT)). TICT is observed in many molecular systems, including the retinal chromophore, and is highly modulated by the polarity of the surrounding environment.^68−76^ In the higher electronic state, the TICT state allows the photoexcited molecule to return to the ground state with higher efficiency. The difference in charge distribution between fragments along the photoisomerization coordinate (Figure S1B) shows that the charge distribution between fragments remains generally unaltered along the isomerization pathway. In the case of the retinal PSB, a similar pattern of charge delocalization is also observed in S_1_, but in S_2_ the charge distribution was calculated to be similar to S_0_.^77^ This difference in charge delocalization in the S_2_ state of BP is possibly due to a more resonant π-system resulting from the presence of a phenyl ring instead of the β-ionone ring of retinal.

We then generated the 2D potential energy surfaces of the S_0_ and S_1_ states around the MECI_*E*/*Z*_ point by employing the orthonormalized gradient difference (GDV) and nonadiabatic coupling vectors (NACV), which correspond to BLA and torsional motion coordinates, respectively (Figure S2A,B). The 2D scan produced a peaked conical intersection without any discontinuity (Figure S2C). A smooth topology around the MECI point validates the applicability of the XMS-CASPT2 method near the surface crossing region. The appearance of a peaked conical intersection is validated by plotting the S_0_ and S_1_ energies centered around the minimum energy conical intersection for varying circular loop angles (Figure S2D). The circular path shows that the S_0_ and S_1_ energies do not cross the energy of the minimum energy conical intersection. Moreover, there is only one minimum in the S_0_ energy surface and therefore the intersection can be classified as a peaked single-path intersection.

Therefore, photoisomerization of BP occurs through a peaked conical intersection, which is energetically more accessible from the stable *E* isomer compared to the *Z* isomer. This peaked MECI aids in effectively steering the excited state population away from the intersection toward the ground state conformers, thereby facilitating the formation of photoproduct or regeneration of the initial state. This phenomenon is highly desirable in photoswitches, highlighting the potential utility of BP in such applications. However, in the case of more complex conjugated molecules, achieving the desired isomerization around a particular double bond is more challenging.

### 3.2 Investigation of ExBP

#### 3.2.1 Excitation Energies

**Scheme 1:**
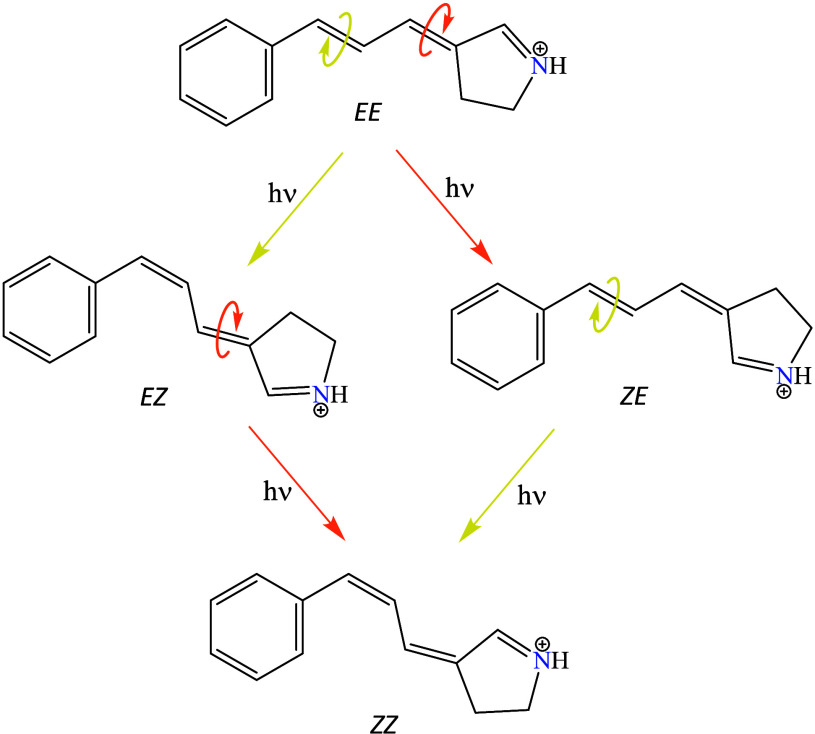
Possible Photoisomers of ExBP Generated by Successive Rotations of Each of the Two Intercyclic Conjugated Double Bonds The direction of the rotation is arbitrary.

Introduction of an additional conjugated double bond in the intercyclic unit of BP generates ExBP. The investigation of ExBP provides an opportunity to explore several photoisomerization pathways resulting from sequential or reversible rotations around two double bonds, leading to the formation of diverse photoproducts. Specifically, photoisomerization of ExBP can result in four possible configurations due to the possible successive rotations of the two intercyclic conjugated double bonds, as shown in Scheme 1. These configurations are (3*E*,7*E*), (3*Z*,7*E*), (3*E*,7*Z*), and (3*Z*,7*Z*); for simplicity, they will be named *EE*, *ZE*, *EZ*, and *ZZ* throughout the manuscript.

The geometries of all the configurations in the ground state were optimized using the XMS-CASPT2/cc-pVDZ level of theory. The stability of the isomers follows the order of *EE* (0) > *EZ* (0.09 eV) > *ZE* (0.12 eV) > *ZZ* (0.19 eV). Table 1 lists the calculated excitation energies of the isomers by employing the XMS-CASPT2/cc-pVDZ methodology on optimized geometries. Tables S3 and S4 provide excitation energies using various methods with the cc-pVDZ and cc-pVTZ basis sets, respectively. Similarly to BP, the first excited state (S_1_) of all the ExBP isomers is optically bright at all levels of theories considered here. The relative brightness of S_1_ for various isomers follows the order *EE* > *ZE* > *EZ* > *ZZ*. The variations in relative brightness among these isomers can be attributed to the extent of structural twisting and deviation from planarity. The brightness of the second and third excited states of ExBP is insignificant; however, the third excited state (S_3_) is comparatively brighter than the second excited state (S_2_), as observed for BP. The S_2_ excited state is well separated from the S_1_ ranging in 0.53–0.75 eV at the XMS-CASPT2/cc-pVDZ and 0.53–0.92 eV considering CC2/cc-pVDZ and ADC(2)/cc-pVDZ methodologies. The excitation energies using the cc-pVTZ basis set provide quantitatively similar values to those obtained with the cc-pVDZ basis set.

**Table 1:** Vertical Excitation Energies and Oscillator Strengths (*f*) for Different Isomers of ExBP Using the XMS-CASPT2/cc-pVDZ Methodology

excitation	*EE*		*ZE*		*EZ*		*ZZ*	
	Δ*E*_calc_, eV (nm)	*f*	Δ*E*_calc_, eV (nm)	*f*	Δ*E*_calc_, eV (nm)	*f*	Δ*E*_calc_, eV (nm)	*f*
S_0_ → S_1_	2.93 (423)	1.58	2.83 (438)	1.24	3.19 (389)	0.82	3.15 (393)	0.74
S_0_ → S_2_	3.58 (346)	0.01	3.58 (347)	0.01	3.72 (333)	0.00	3.78 (328)	0.00
S_0_ → S_3_	4.00 (310)	0.05	3.99 (311)	0.12	4.13 (300)	0.29	4.10 (303)	0.19

#### 3.2.2 Photoisomerization Paths

After absorbing a photon, a given ExBP isomer can in principle lead to two different photoproducts depending on the selective rotation around a particular double bond; however, one pathway can be photochemically preferred over another. The photoisomerization paths connecting the four different isomers of ExBP were analyzed using XMS-CASPT2/cc-pVDZ methodology. The paths include the MECI between the optically bright first excited state (S_1_) and the ground state (S_0_). The geometries of the isomerization profile were obtained by the LIIC method.

##### 3.2.2.1 *EE* → *ZE* vs *EE* → *EZ* Isomerization

Among the four possible configurations of ExBP, the *EE* isomer is energetically most stable in the ground electronic state. Starting from the *EE* form, two isomers can be generated by rotation around each of the two intercyclic double bonds. Rotation of the double bond closer to the locked Schiff base leads to the formation of the *ZE* isomer, while rotation around the double bond closer to the phenyl ring results in the *EZ* isomer, as shown in Scheme 1.

**Figure 4:**
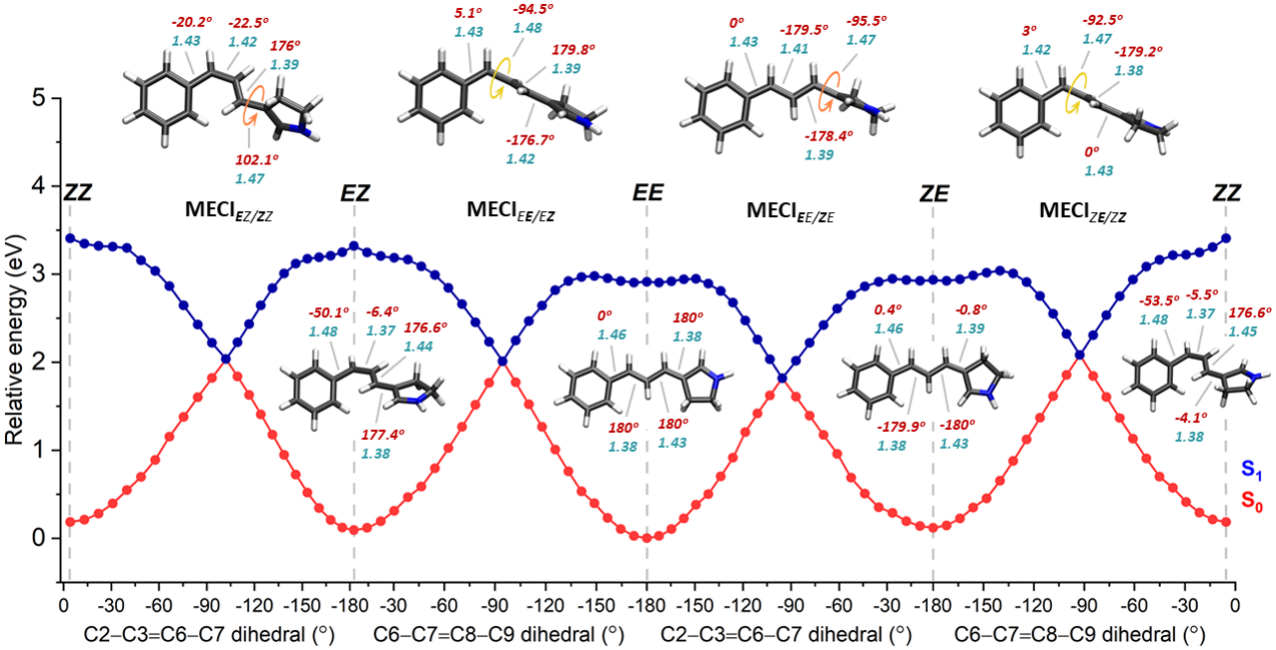
Photoisomerization profiles due to the successive rotation of the two intercyclic conjugated double bonds of ExBP and minimum energy structures. The dihedral and bond length values are shown in red and turquoise, respectively.

The *EE* isomer of the ExBP molecule is planar in the ground state, with dihedrals around the intercyclic double bonds φ_3=6_ (C2–C3=C6–C7) and φ_7=8_ (C6–C7=C8–C9) of approximately 180 degrees. During the *EE* to *ZE* isomerization (***E****E* → ***Z****E*), while the φ_7=8_ dihedral remains unchanged, the φ_3=6_ dihedral isomerizes to about zero degrees. However, in the case of *EE* to *EZ* isomerization (*E****E*** → *E****Z***), the molecule undergoes a more profound structural transformation. In the *EZ* configuration, the φ_7=8_ dihedral adopts a *Z*-configuration with a twist of approximately −6.4 degrees. In addition, a significant deformation was observed around the C7=C8–C9=C10 (φ_8–9_) dihedral of about −50.1 degrees due to the steric interaction of phenyl ring hydrogen with the hydrogen atom attached to the C6 atom. These structural characteristics along with photoisomerization profiles are shown in Figure 4.

During ***E****E* → ***Z****E* isomerization, the S_1_ excited state potential energy surface remains relatively flat with only a small barrier of 0.04 eV, facilitating a smooth transition between S_1_ and S_0_ states. This process passes through a MECI_***E****E*/***Z****E*_ where the φ_3=6_ dihedral is twisted to approximately −95.5 degrees. Meanwhile, the nearby single bond C3=C6–C7=C8 (φ_6–7_) remains mostly unaltered which signifies a one-bond-flip (OBF) mechanism. At the MECI_***E****E*/***Z****E*_ point, a small pyramidalization of 2.3 degrees was observed at the nitrogen atom. During isomerization, bond length alternation (BLA; extension of double bonds and contraction of single bonds) was observed. The BLA was calculated as the difference between the average single bond length and the average double bond length of the π-conjugated system. The BLA was found to be 0.06 for *EE*, 0.03 for MECI_***E****E*/***Z****E*_, and 0.07 Å for *ZE*, considering only the intercyclic conjugated system with C3=C6/C7=C8 double bonds and C6–C7/C8–C9 single bonds.

The *EE* form can also isomerize to *EZ*. The *E****E*** → *E****Z*** isomerization encounters an energy barrier of 0.07 eV in the S_1_ state leading to the MECI_*E****E***/*E****Z***_. This barrier is marginally higher (about 0.03 eV) than the energy barrier observed for the ***E****E* → ***Z****E* isomerization, which is primarily due to the structural strain introduced by the rotation of the double bond near the bulky phenyl ring in the *EZ* isomer. At the MECI_*E****E***/*E****Z***_, the φ_7=8_ dihedral angle is twisted to approximately −94.5 degrees, accompanied by a minor concomitant twist of the φ_8–9_ dihedral by about 5 degrees (OBF photoisomerization mechanism). The BLA values are 0.06 for *EE*, 0.04 for MECI_*E****E***/*E****Z***_, and 0.09 Å for *EZ*. The relatively larger BLA in the *E****E*** → *E****Z*** isomerization can also be explained by the twist in the *EZ* configuration, resulting in a less conjugated π-system and more defined bond orders.

**Figure 5:**
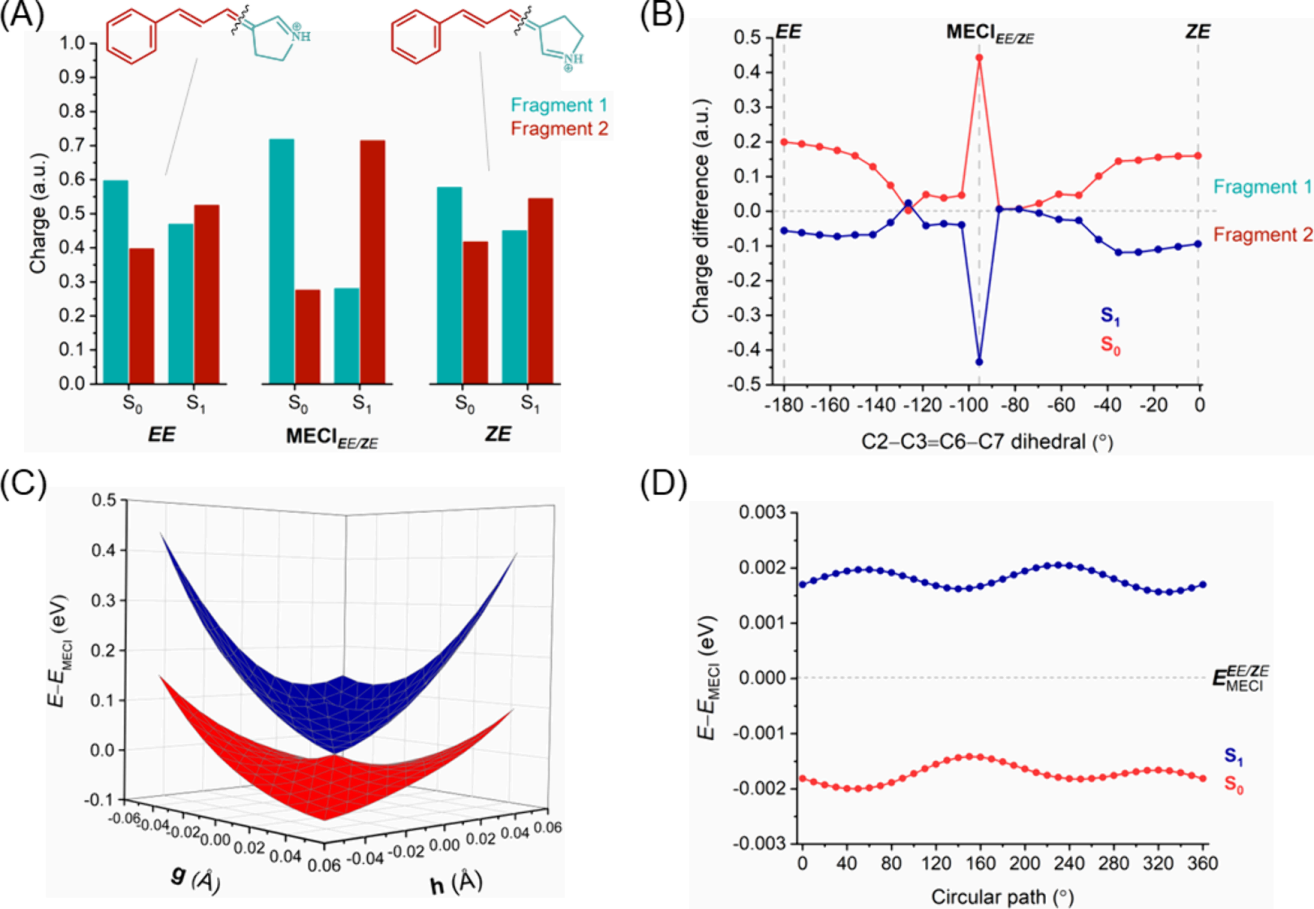
***E****E* → ***Z****E* isomerization. (A) Charge distribution on the molecular fragments of ExBP at each side of the isomerized double bond (C3=C6) for the *EE* and *ZE* isomers and MECI_***E****E/****Z****E*_ point in S_0_ and S_1_. (B) Difference in charge distribution between fragments (i.e., “fragment 1 charge” minus “fragment 2 charge”) along the photoisomerization pathway. The horizontal line in the *y*-axis separates the charges between the two molecular fragments, with positive values indicating a larger positive charge in fragment 1 and vice versa. (C) 2D potential energy surfaces (in eV) of S_0_ and S_1_ along two branching vectors (GDV; **g** and NACV; **h**) centered around minimum energy conical intersection. (D) Plot of S_0_ and S_1_ energies (in eV) centered around the minimum energy conical intersection for a varying circular loop angle.

**Figure 6:**
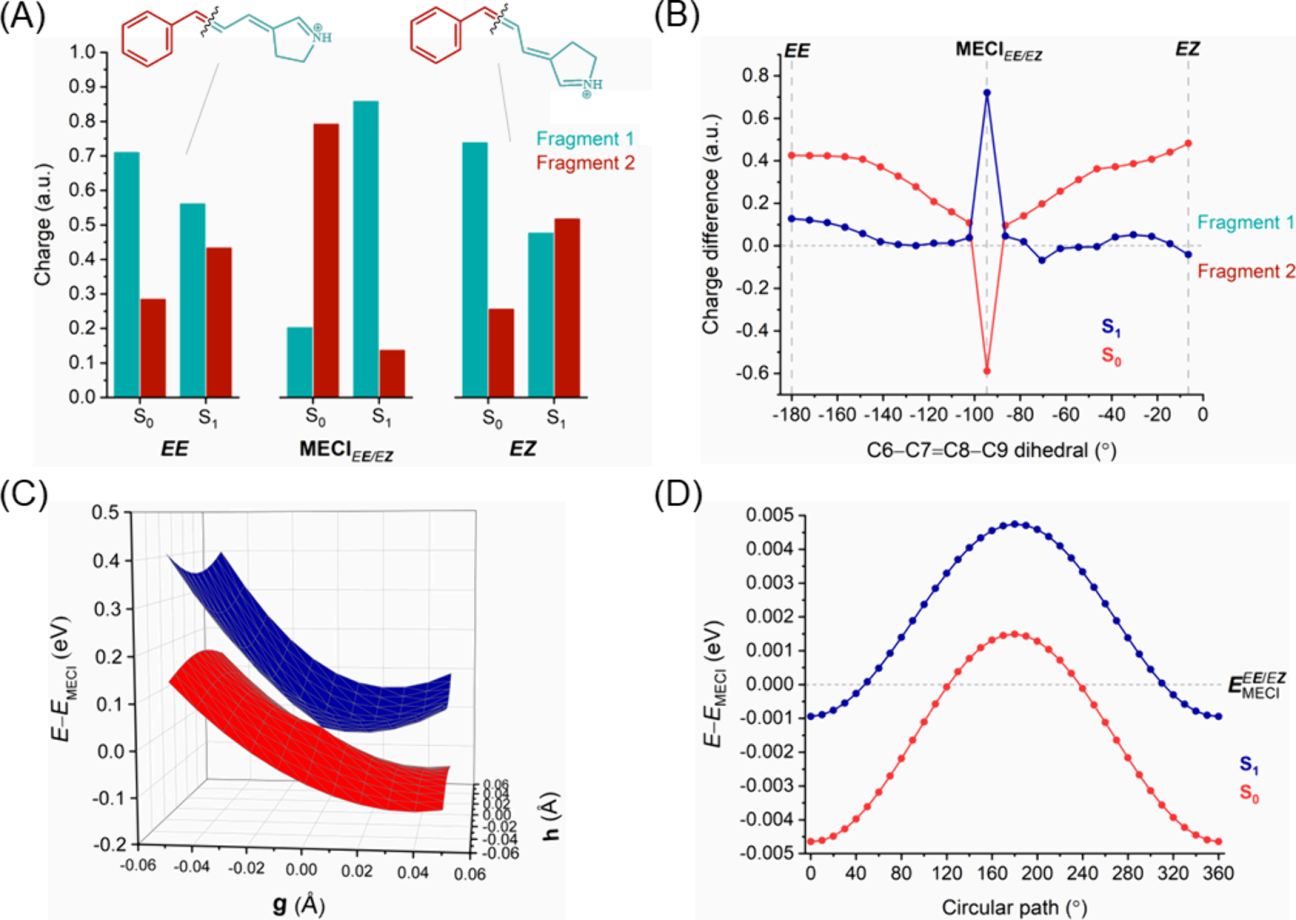
*E**E*** → *E****Z*** isomerization. (A) Charge distribution on the molecular fragments of ExBP at each side of the isomerized double bond (C7=C8) for the *EE* and *EZ* isomers and MECI_*E****E****/E****Z***_ point in S_0_ and S_1_. (B) Difference in charge distribution between fragments (i.e., “fragment 1 charge” minus “fragment 2 charge”) along the photoisomerization pathway. The horizontal line in the *y*-axis separates the charges between the two molecular fragments, with positive values indicating a larger positive charge in fragment 1 and vice versa. (C) 2D potential energy surfaces (in eV) of S_0_ and S_1_ along two branching vectors (GDV; **g** and NACV; **h**) centered around minimum energy conical intersection. (D) Plot of S_0_ and S_1_ energies (in eV) centered around the minimum energy conical intersection for a varying circular loop angle.

The redistribution of the molecular charges during photoisomerization was investigated by dividing the molecule into two fragments separated at the isomerizing C3=C6 (for ***E****E* → ***Z****E*) and C7=C8 (for *E****E*** → *E****Z***) bonds. Figures 5A and 6A show the distribution of S_0_, and S_1_ charges in the two fragments for the ***E****E* → ***Z****E* and *E****E*** → *E****Z*** isomerizations, respectively. Comparison with S_2_ charges is shown in Figure S3. In both cases, in the initial S_0_ state of *EE*, the positive charge is concentrated near the Schiff base region (fragment 1), which delocalizes to fragment 2 in the excited states. In S_2_ the charge delocalization is generally higher than the S_1_, possibly due to a stronger stabilization by the phenyl ring. However, at the MECIs of the two possible isomerization routes, ***E****E* → ***Z****E* or *E****E*** → *E****Z***, the charge distribution between the two fragments differs. In the ***E****E* → ***Z****E* isomerization, the MECI_***E****E/****Z****E*_, has a similar charge distribution to that of initial and photoproduct isomers (*EE* and *ZE*), albeit larger in magnitude. However, in the *E****E*** → *E****Z*** isomerization, the MECI_*E****E****/E****Z***_ has a reversed charge distribution, with most of the positive charge accumulating in the benzylidene fragment. Compared to BP, the charge delocalization between fragments at the MECIs is higher in ExBP, especially in the S_0_ and S_1_ states.

The difference in charges between fragments along photoisomerization pathway in S_0_ and S_1_ states (Figures 5B and 6B for the ***E****E* → ***Z****E* and *E****E*** → *E****Z*** isomerizations, respectively) provides a clearer representation of the dynamic interplay between electronic states. The charge difference plots exhibit an out-of-phase oscillatory relationship between the S_0_ and S_1_ states, signifying a robust electronic coupling between these two states.^78,79^ Interestingly, in the *E****E*** → *E****Z*** isomerization route this coupling results in an exchange of electronic (and charged) characters between S_0_ and S_1_ at the MECI_*E****E****/E****Z***_ point (Figure 6B), which is not observed in the case of MECI_***E****E/****Z****E*_ (Figure 5B).

The 2D scans of the potential energy surface for the S_0_ and S_1_ states along the branching plane vectors (GDV and NACV) around the conical intersections (MECI_***E****E/****Z****E*_ and MECI_*E****E****/E****Z***_) of the two possible isomerization routes are shown in Figures 5C and 6C, respectively. The branching plane vectors are given in Figures S4 and S5, respectively. The topology parameters for these MECIs were calculated and are listed in Table S5. The topography of the potential energy surfaces is clearly different in the two routes, which results in different photodynamic properties. In ***E****E* → ***Z****E* isomerization, with the isomerizing double bond closer to the Schiff base, the topography of MECI_***E****E/****Z****E*_ is peaked. On the other hand, in *E**E*** → *E****Z*** isomerization, further from the Schiff base, the topography of MECI_*E****E****/E****Z***_ is sloped. This difference in the topography of the potential energy surfaces can also be observed in the S_0_ and S_1_ energies along the circular loop centered around the conical intersections for the two possible isomerization pathways of *EE*, as shown in Figures 5D and 6D, respectively. The circular path energies around MECI_***E****E/****Z****E*_ do not cross the energy of the conical intersection and feature two minima, which signifies that the intersection is peaked and bifurcated. On the other hand, the circular path energies around MECI_*E****E****/E****Z***_ do cross the energy of the conical intersection and have only one minimum; therefore, this intersection can be classified as a sloped single-path. Furthermore, we have plotted the charge differences between molecular fragments along the circular loop around the MECI for ***E****E* → ***Z****E* and *E****E*** → *E****Z*** isomerizations (Figure S8). For both isomerizations, although the charge difference at the MECI is very large, it exhibits an oscillatory behavior between smaller and larger differences along the circular loop.

In summary, the ***E****E* → ***Z****E* isomerization in ExBP closely resembles that observed in the simpler BP model, as both involve a twist around the double bond near the Schiff base. Consequently, the charge distributions in the molecular fragments and the MECI characteristics (topology and topography) follow similar patterns. The *EE* configuration of ExBP has a lower energy barrier and a more stable MECI when photoisomerizing to the *ZE* isomer, compared to *EZ*. In addition, the topography near the MECI_***E****E*/***Z****E*_ is peaked, while for the MECI_*E****E***/*E****Z***_ it is sloped. A peaked intersection is more effective in directing the excited population to the ground state, whereas a sloped intersection can up-funnel the population back to the excited state, thereby reducing the probability of photoproduct formation. Thus, starting from the *EE* isomer, photoisomerization leads preferably to the formation of the *ZE* isomer, energetically and topographically preferred, instead of transitioning to the *EZ* isomer. This difference in preferred product formation possibly arises from the structural peculiarities of ExBP that hinder the rotation of the molecular unit adjacent to the bulky phenyl ring, rendering the *EZ* photoisomerization path less favorable. A similar phenomenon can be observed in the retinal chromophore of rhodopsins, as isomerization around double bonds further from the Schiff base and near the β-ionone ring – leading to 7-cis or 9-cis configurations – is relatively rare.

##### 3.2.2.2 *EE* ← *ZE* vs *ZE* → *ZZ* Isomerization

In the previous section, we explored the two possible isomerization routes from the *EE* isomer to the *ZE* and *EZ* photoproducts. In multiphoton excitation, such photoproducts can be further excited, leading either to a “sequential” isomerization to form the *ZZ* isomer, or to a “reversed” isomerization back to *EE*.

Starting from the *ZE* isomer (the most favorable photoproduct resulting from *EE* photoexcitation), the reaction can either isomerize back to the initial *EE* form (***E****E* ← ***Z****E*) by rotating around the φ_3=6_ dihedral, or alternatively form the photoproduct *ZZ* (*Z**E*** → *Z****Z***) by undergoing isomerization around the φ_7=8_ dihedral. The *ZE* and *EE* isomers of the first reaction are planar, while the *ZZ* isomer in the second reaction is highly distorted mainly due to the highly twisted φ_8–9_ dihedral measuring approximately −53.5 degrees. These asymmetries of the structural transitions in the two possible isomerizations are reflected in the energetic barriers in the S_1_ state leading to each conical intersection (Figure 4), 0.02 eV for ***E****E* ← ***Z****E* back to the original isomer and an appreciably higher 0.12 eV for the sequential *Z****E*** → *Z****Z*** reaction. In this latter case, at the MECI_*Z****E****/Z****Z***_ the φ_7=8_ dihedral is twisted to approximately −92.5 degrees, while the φ_8–9_ dihedral angle is around 3 degrees, signifying a one-bond-flip (OBF) mechanism of the isomerization. A larger BLA for this sequential *Z****E*** → *Z****Z*** reaction (0.07 for *ZE*, 0.05 for MECI_*Z****E****/Z****Z***_, and 0.09 Å for *ZZ*) also indicates significant structural changes during photoisomerization (see Section 3.2.2.1).

**Figure 7:**
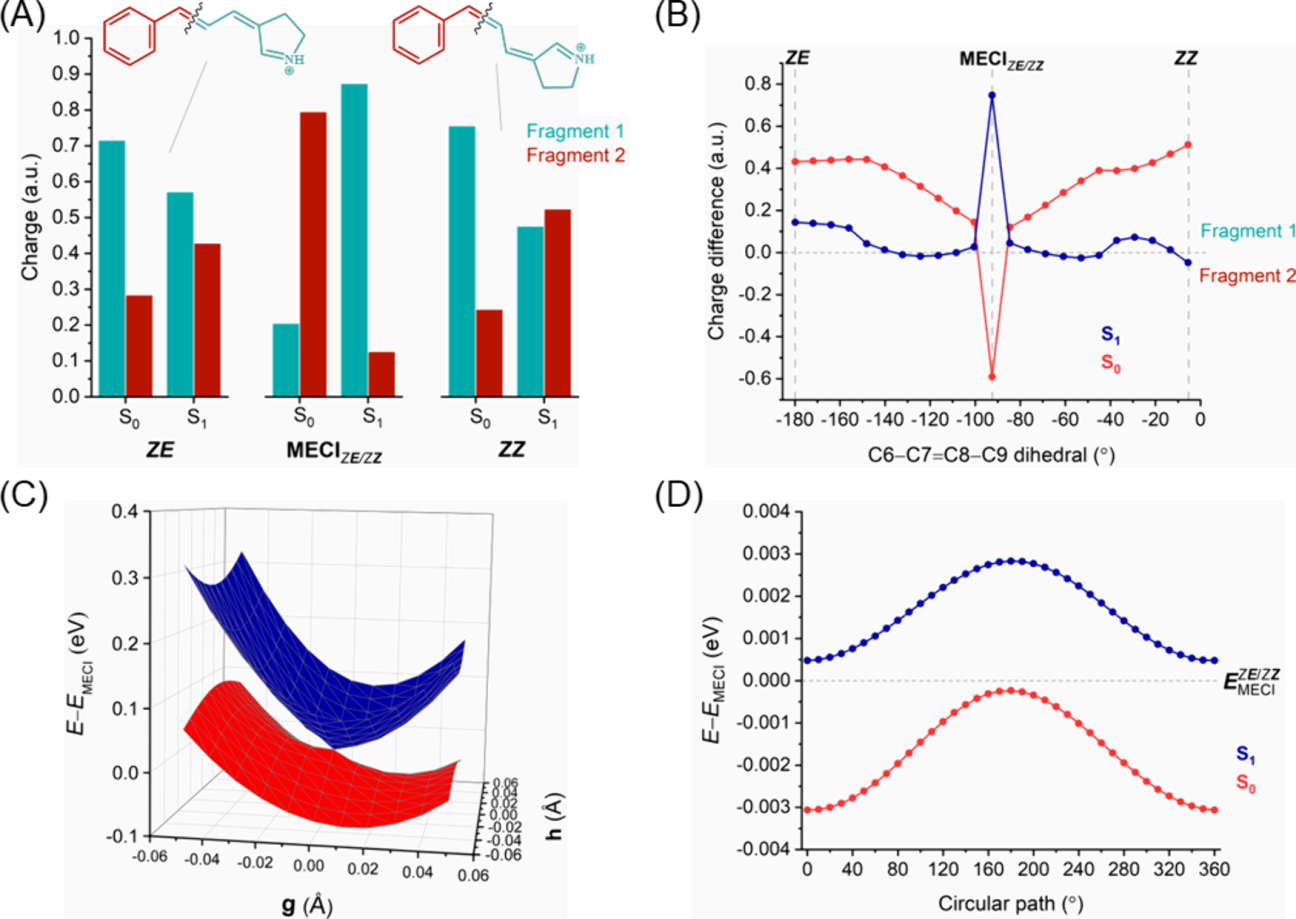
*Z**E*** → *Z****Z*** isomerization. (A) Charge distribution on the molecular fragments of ExBP at each side of the isomerizing double bond (C7=C8) for the *ZE* and *ZZ* isomers and MECI_*Z****E****/Z****Z***_ point in S_0_ and S_1_. (B) Difference in charge distribution between fragments (i.e., “fragment 1 charge” minus “fragment 2 charge”) along the photoisomerization pathway. The horizontal line in the *y*-axis separates the charges between the two molecular fragments, with positive values indicating a greater positive charge in fragment 1 and vice versa. (C) 2D potential energy surfaces (in eV) of S_0_ and S_1_ along two branching vectors (GDV; **g** and NACV; **h**) centered around the minimum energy conical intersection. (D) Plot of S_0_ and S_1_ energies (in eV) centered around the minimum energy conical intersection for a varying circular loop angle.

The intramolecular charge distribution of the *Z****E*** → *Z****Z*** isomerization was analyzed by dividing ExBP into two fragments separated at the isomerizing C7=C8 double bond (Figure 7A). For the S_0_ ground state of both *ZE* and *ZZ* isomers, the positive charge is localized near the Schiff base region (fragment 1) and delocalizes toward the benzylidene ring (fragment 2) in the excited states S_1_ and S_2_ (Figure S3). The charge difference plot (Figure 7B) reveals the robust electronic coupling between these between the S_0_ and S_1_ states. Interestingly, this coupling results in the exchange of electronic characters between S_0_ and S_1_ at the MECI_*Z****E****/Z****Z***_, as observed in the case of MECI_*E**E**/E****Z***_ (Figure 6B). Both of these isomerizations (*E****E*** → *E****Z*** and *Z****E*** → *Z****Z***) entail a rotation of the C7=C8 double bond. In the *Z****E*** → *Z****Z*** reaction, the 2D potential energy surfaces for the S_0_ and S_1_ in the vicinity of the MECI_*Z****E****/Z****Z***_ point have a distinctive peaked conical intersection (Figures 7C and S6). The circular path energies (Figure 7D) suggest that this intersection, with only one minimum in the circular path, can be classified as a peaked single-path. On the other hand, the MECI_***E****E/****Z****E*_ in the ***E****E* ← ***Z****E* isomerization has a peaked bifurcating intersection (see Section 3.2.2.1).

In summary, the *Z****E*** → *Z****Z*** isomerization in ExBP is hindered by several factors and, hence, photoexcitation of the *ZE* isomer will preferably revert it back to the *EE* configuration, rather than transitioning to the *ZZ* isomer.

##### 3.2.2.3 *EE* ← *EZ* vs *EZ* → *ZZ* Isomerization

The *EZ* isomer of ExBP is the least favorable product resulting from the photoexcitation of the stable *EE* isomer, as discussed in Section 3.2.2.1.

The *EZ* isomer can either photoisomerize back to the initial *EE (E****E*** ← *E****Z***) by rotating around the φ_7=8_ dihedral or form *ZZ* by undergoing photoisomerization around the φ_3=6_ dihedral. The *EE* isomer is planar while both *EZ* and *ZZ* isomers are twisted at the φ_8–9_ dihedral. Formation of either photoproduct, *EE* or *ZZ*, from *EZ* is favorable as there are no energy barriers in the S_1_ state for these reactions. The MECI_***E****Z/****Z****Z*_ of the ***E****Z* → ***Z****Z* isomerization is located at a φ_3=6_ dihedral of about −102 degrees and presents a considerable twist of the φ_7=8_ (∼16°) and the φ_8–9_ (∼30°) dihedrals compared to *EZ* isomer, even though φ_6–7_ remains unchanged. Thus, the isomerization around the central double bond is associated with partial rotations of other single and double bonds. Also, we observed a pyramidalization at the nitrogen atom of 5.7°. As expected, such pronounced structural changes in the ***E****Z* → ***Z****Z* isomerization are associated with relatively high BLA values (0.09 for *EZ*, 0.03 for MECI_***E****Z/****Z****Z*_, and 0.09 Å for *ZZ*).

**Figure 8:**
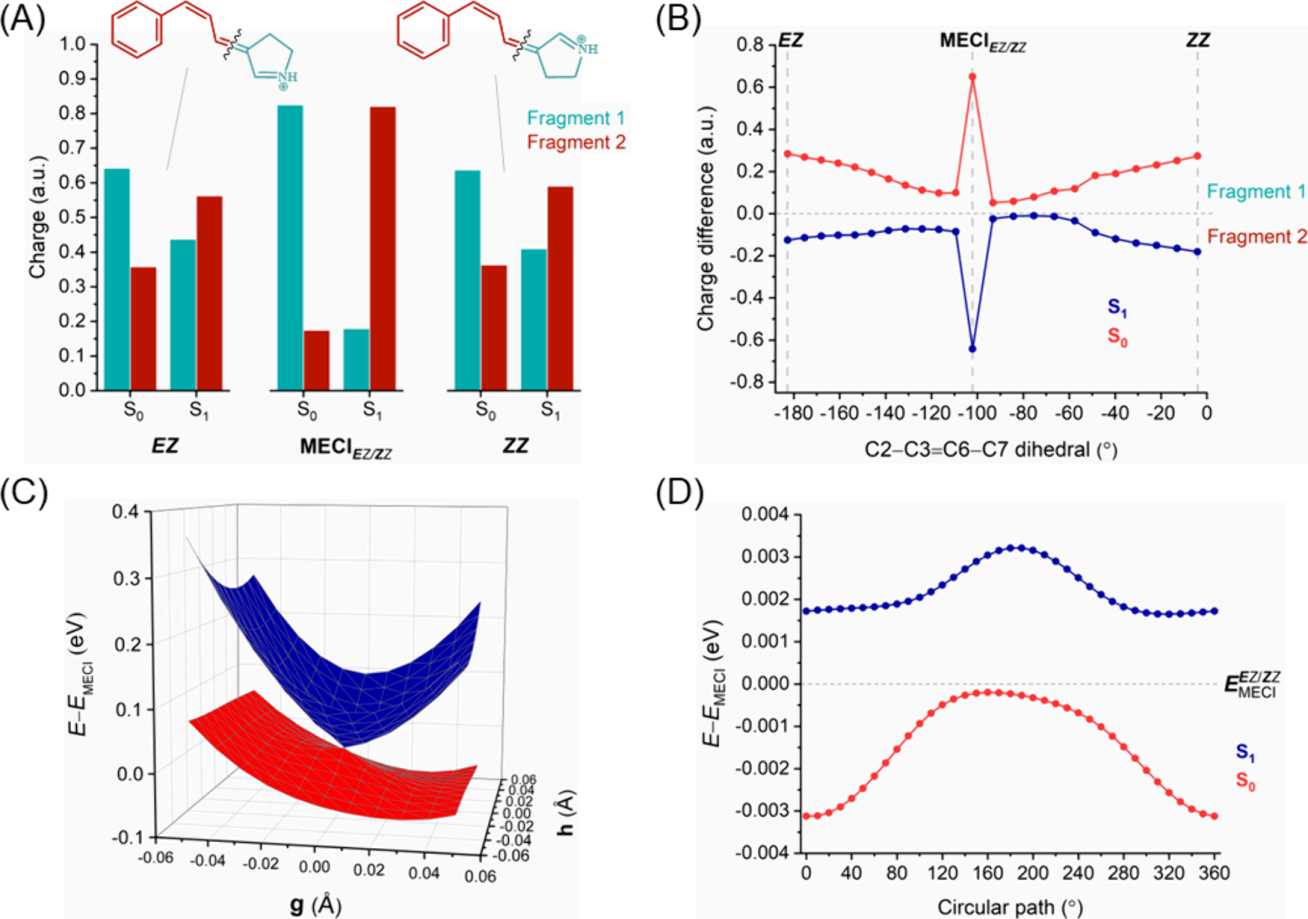
***E****Z* → ***Z****Z* isomerization. (A) Charge distribution on the molecular fragments of ExBP at each side of the isomerizing double bond (C7=C8) for the *EZ* and *ZZ* isomers and MECI_***E****Z/****Z****Z*_ point in S_0_ and S_1_. (B) Difference in charge distribution between fragments (i.e., “fragment 1 charge” minus “fragment 2 charge”) along the photoisomerization pathway. The horizontal line in the *y*-axis separates the charges between the two molecular fragments, with positive values indicating a greater positive charge in fragment 1 and vice versa. (C) 2D potential energy surfaces (in eV) of S_0_ and S_1_ along two branching vectors (GDV; **g** and NACV; **h**) centered around the minimum energy conical intersection. (D) Plot of S_0_ and S_1_ energies (in eV) centered around the minimum energy conical intersection for a varying circular loop angle.

For ***E****Z* → ***Z****Z*, the charge distribution pattern of the molecular fragments is similar to ***E****E* → ***Z****E* (see Section 3.2.2.1), both involving isomerization of the C3=C6 double bond. The positive charge is localized in fragment 1 in the ground state, while it is shifted to fragment 2 in excited states (Figure 8A). The magnitude of charge separation is higher at MECI_***E****Z/****Z****Z*_ point. The charge difference along the photoisomerization pathway (Figure 8B) shows an out-of-phase coupling between S_0_ and S_1_ – especially at the MECI_***E****Z/****Z****Z*_ – but without an exchange of electronic characters, as observed in the ***E****E* → ***Z****E* isomerization (Figure 5B). The 2D potential energy surfaces (Figures 8C and S7) and circular path energies (Figure 8D) for the S_0_ and S_1_ electronic states along branching plane vectors around MECI_***E****Z/****Z****Z*_ feature a peaked single-path conical intersection. In contrast, the MECI for the alternative isomerization (*E****E*** ← *E****Z***) pathway (MECI_*E****E****/E****Z***_) exhibits a sloped single-path topography (Figure 6C,D).

## 4 Conclusions

Selectivity of isomerization in linear conjugated chromophores (i.e., which double bond in the system is isomerized upon photoexcitation) is an area of dynamic ongoing research, as we cannot yet fully predict or control it. In the absence of any external influence, factors intrinsic to the chromophore may establish some inherent selectivity. Inside a protein environment, this inherent selectivity can be then modulated by various factors, such as steric and electrostatic interactions with nearby amino acids, ions or other cofactors, and water molecules. In this article, we investigate the intrinsic factors that establish isomerization selectivity in two simple models of the retinal PSB, BP and ExBP, with one and two double bonds that can undergo photoisomerization. Using advanced quantum mechanics calculations to accurately treat electronic effects in photoexcited states, we have characterized each possible photoisomerization reaction of BP and ExBP by measuring electronic energies, charge distribution, and the topology and topography of the minimum energy conical intersections. We focus our discussion on photochemical selectivity on ExBP, where the potential for rotation around two double bonds presents challenges similar to those existing in other conjugated chromophores, such as the retinal PSB.

**Figure 9:**
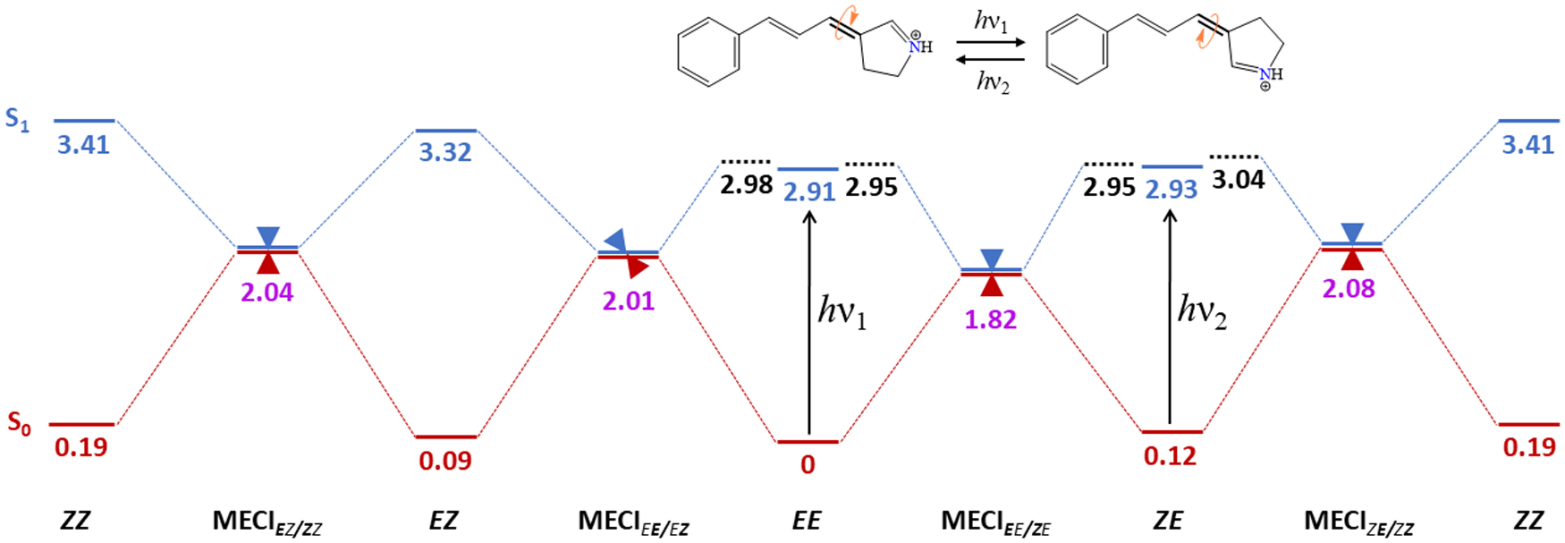
Energy landscape of ExBP photoisomerization. Energies are reported in eV with respect to the ground state energy of the *EE* isomer (S_0_ in red, and S_1_ in blue). The black dotted lines represent the energy barriers in the photoisomerization path. The S_1_/S_0_ MECIs are represented by cones (peaked and sloped). The preferred isomerization pathway is the back and forth reaction ***E****E* ↔ ***Z****E* represented schematically at the top of the figure.

The energy landscape for all possible isomerizations of ExBP is illustrated in Figure 9. Photoexcitation (*h*ν_1_) of the most stable *EE* configuration can lead to two isomers, *ZE* or *EZ*, but there are several factors that favor isomerization of the double bond near the Schiff base (C3=C6) to form the *ZE* isomer. In the S_1_ excited state (blue) of *EE*, the pathway leading to *ZE* has both a slightly lower barrier (0.03 eV) and a more stable conical intersection (0.19 eV) of peaked bifurcating topography. A peaked intersection acts as a funnel, while a bifurcation allows two preferred paths for relaxation leading to or from the intersection, resulting in a fast transition to the ground state. Conversely, isomerization to *EZ* involves crossing a sloped single-path conical intersection that requires a continuous descent toward the intersection that can take longer to reach. Moreover, a sloped intersection allows a transition back to the excited state – rather than leading to the ground state – that also results in longer excited-state lifetimes. On the other hand, in this single-path intersection, there is only one relaxation pathway to the ground state. The two minima in the circular loop around the MECI for the bifurcating ***E****E* ↔ ***Z****E* isomerization in the S_1_ are dominated by the BLA coordinate, while the minima in the S_0_ are governed by the dihedral torsion components (Figure 5D). However, in the case of *E****E*** ↔ *E****Z*** isomerization, both S_0_ and S_1_ are relaxed along the BLA coordinate (Figure 6D). Even though the initial relaxation occurs along the BLA coordinate, the torsional motion should effectively lead to the isomerized products in the ground state. While these differences in the topography of the conical intersection affect photodynamics, they can only be meaningfully discussed in the close vicinity of the conical intersection. In general, ExBP, as a linear conjugated model of retinal PSB, prefers to isomerize around the double bond closer to the benzylidene ring, thereby resembling the retinal PSB of microbial rhodopsins that undergo isomerization from all-*trans* to the 13-*cis* configuration. When the main photoproduct *ZE* is further excited (*h*ν_2_), it prefers to revert to the initial *EE* configuration as isomerization to the energetically unfavorable *ZZ* isomer requires crossing a considerably higher energy barrier to the MECI_*Z****E****/Z****Z***_. Thus, multiple photoexcitation of ExBP results preferentially in a back-and-forth isomerization between the *EE* and *ZE* forms, rather than in sequential isomerization of different double bonds (Figure 9). This ability to switch between two main forms is a highly desirable property in photoswitches. Such bistability is also observed in the retinal PSB of some rhodopsins. While this topic is beyond the scope of this paper, we speculate that bistability in the retinal PSB is also inherent to the molecule rather than conferred by the protein environment.

**Figure 10:**
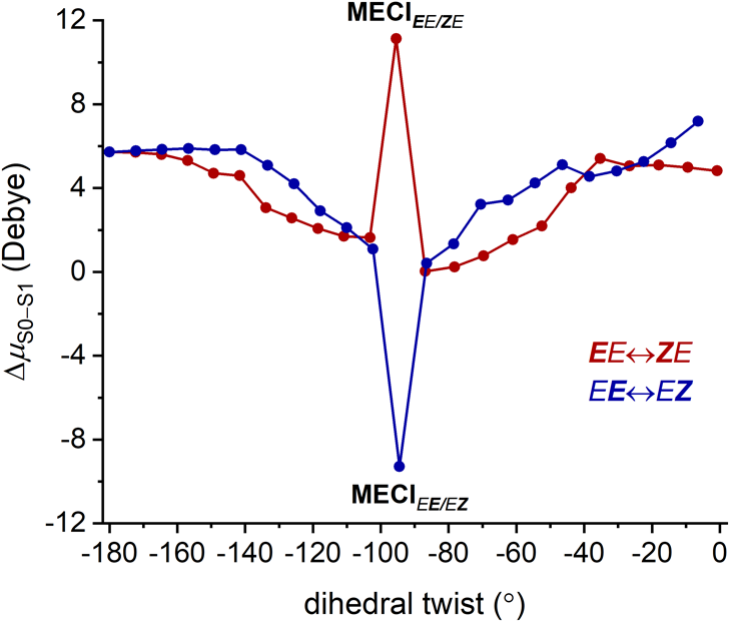
Dipole moment difference between ground and first excited states (Δ*μ*_S0–S1_) for ***E****E* ↔ ***Z****E* and *E****E*** ↔ *E****Z*** isomerizations.

One intriguing observation in our analysis is the opposite charge distribution at the minimum energy conical intersections in the isomerization around C3=C6, closer to the pyrroline (***E****E* ↔ ***Z****E*), and around C7=C8, further away (*E****E*** ↔ *E****Z***) (Figure S9). This effect can also be observed in the difference in dipole moments between ground and excited states (Δ*μ*_S0–S1_) (Figure 10).

In the ground state of ExBP, the positive charge resides primarily near the Schiff base. In the S_1_ excited state, the positive charge delocalizes to the polyene. Along the photoisomerization pathway, the dipole of S_1_ progressively approaches that of S_0_. However, the difference in dipole moment peaks at the S_1_/S_0_ conical intersection where the structural deformation is maximum. This phenomenon has also been observed in other retinal PSB analogs.^77,80,81^ For ExBP, depending on the isomerizing double bond, the altered electron distribution between the S_0_ and S_1_ states results in opposite dipole moments at the conical intersection. These observed striking differences at the conical intersections can affect the photodynamics of its possible isomerization routes, as different dipoles will be differently modulated or stabilized by the polarity of the environment, e.g., by solvents.^82^ Furthermore, this redistribution of electrons at the conical intersection can result in new configurations of vibronic states that lead to different relaxation pathways. Such vibronic coupling has been shown to be important in photochemical reactions^83^ and is a critical component of most quantum biological mechanisms.^84^

Our analysis showcases how an intricate interplay between structural and energetic factors and the topography of the conical intersection govern photoisomerization selectivity in highly conjugated chromophores. We expect that our data will provide new and valuable insights into the photodynamics of these fundamental processes.

## Supporting Information Available

Charge distribution in the ground and excited states on the molecular fragments of BP along the photoisomerization pathway; orthonormalized vectors, 2D potential energy surfaces, and the plot of S_0_ and S_1_ energies along circular loop at the MECI for BP; charge distribution on the molecular fragments of ExBP in S_0_, S_1_, and S_2_ states for ***E****E* → ***Z****E*, *E****E*** → *E****Z***, *Z****E*** → *Z****Z***, and ***E****Z* → ***Z****Z* isomerizations; orthonormalized vectors at the MECI for ***E****E* → ***Z****E*, *E****E*** → *E****Z***, *Z****E*** → *Z****Z***, and ***E****Z* → ***Z****Z* isomerizations; difference in charge distribution between molecular fragments along the circular loop around MECI for ***E****E* → ***Z****E* and *E****E*** → *E****Z*** isomerizations; S_0_ and S_1_ atomic charges at the MECI for ***E****E* ↔ ***Z****E* and *E****E*** ↔ *E****Z*** isomerizations; active space orbitals of ExBP used in the XMS-CASPT2 calculations; vertical excitation and emission energies of BP; vertical excitation energies of ExBP using different methodologies with cc-pVDZ and cc-pVTZ basis sets; topology parameters for MECIs of ***E****E* → ***Z****E* and *E****E*** → *E****Z*** isomerizations; and optimized coordinates (PDF)
