# Supplementary material for: Study of Photoselectivity in Linear Conjugated Chromophores Using the XMS-CASPT2 Method
Source: ACS Phys Chem Au. 2024 Oct 2;4(6):736–49. doi: 10.1021/acsphyschemau.4c00065 (PMC11613312; doi:10.1021/acsphyschemau.4c00065)
Supplement: Supplementary file 1 — pg4c00065_si_001.pdf [file pg4c00065_si_001.pdf]

Supporting Information

Study of Photoselectivity in Linear Conjugated Chromophores using the XMS-CASPT2 method

Saumik Sen,*[1,3] and Xavier Deupi*[1,2,3]

1. Condensed Matter Theory Group, Laboratory for Theoretical and Computational Physics, Center for Scientific Computing, Theory, and Data, Paul Scherrer Institute, 5232 Villigen PSI, Switzerland
2. Laboratory of Biomolecular Research, Center for Life Sciences, Paul Scherrer Institute, 5232 Villigen PSI, Switzerland
3. Swiss Institute of Bioinformatics (SIB), 1015 Lausanne, Switzerland

*Corresponding authors

Email: saumik.sen@psi.ch, xavier.deupi@psi.ch

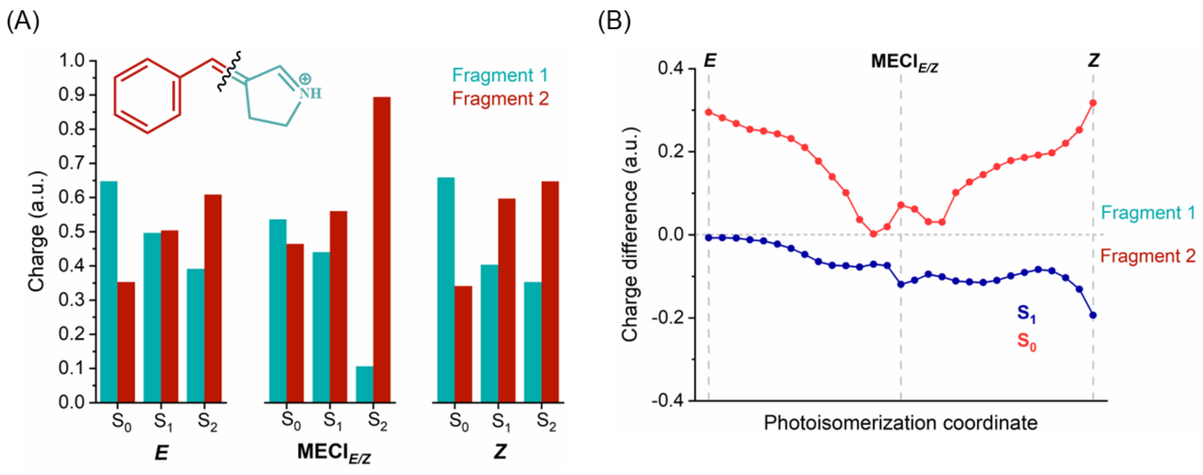

Figure S1. (A) Charge distribution on the molecular fragments of BP at each side of the isomerized central double bond for the *E* and *Z* isomers and MECI$_{E/Z}$ point in S_0, S_1, and S_2. In the ground state (S_0), the positive charge is highly localized on the pyrrolium group mimicking the Schiff base (fragment 1). The charge differences between the fragments (i.e., "fragment 1 charge" minus "fragment 2 charge") are 0.295 a.u. for the *E* isomer, 0.072 a.u. for the MECI$_{E/Z}$ and 0.318 a.u. for the *Z* isomer. In S_1, an electronic charge transfer was observed between the molecular fragments, as in this state the positive charge is more concentrated in fragment 2 comprising phenyl ring with the charge differences of -0.007 a.u. for the *E* isomer, -0.120 a.u. for the MECI$_{E/Z}$ and -0.193 a.u. for the *Z* isomer. A more significant charge migration was observed in the S_2 state where the positive charge is highly localized in fragment 2 (-0.218 a.u. for the *E* isomer, -0.788 a.u. for the MECI$_{E/Z}$ and -0.294 a.u. for the *Z* isomer). **(B)** Difference in charge distribution between fragments along the photoisomerization pathway in S_0 and S_1 states. The horizontal line in the y-axis separates the charges between the two molecular fragments, with positive values indicating a larger positive charge in fragment 1 and vice versa.

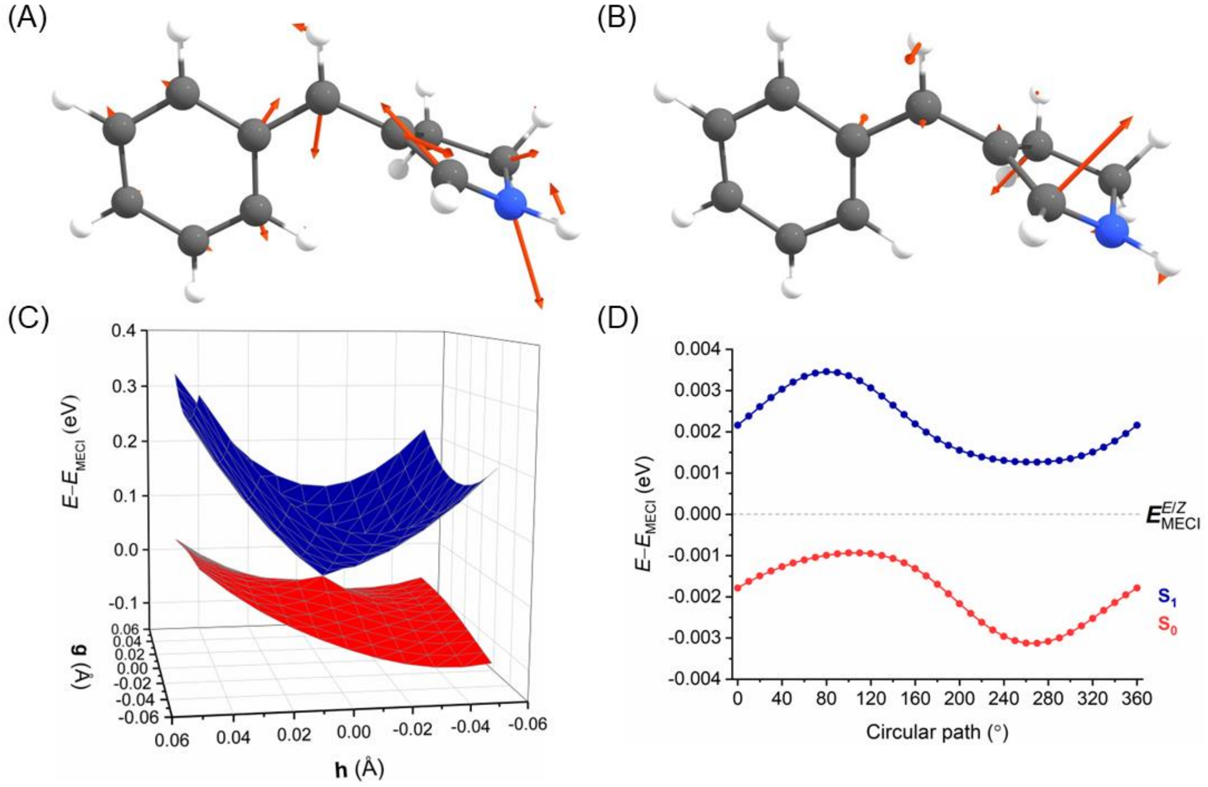

Figure S2. *E*/*Z* isomerization of BP. **(A)** Gradient difference (GDV; **g**) and **(B)** nonadiabatic coupling (NACV; **h**) orthonormalized vectors at the minimum energy conical intersection. **(C)** 2D potential energy surfaces (in eV) of S_0 and S_1 along two branching vectors (GDV; **g** and NACV; **h**) centered around minimum energy conical intersection. **(D)** Plot of S_0 and S_1 energies (in eV) centered around the minimum energy conical intersection for a varying circular loop angle.

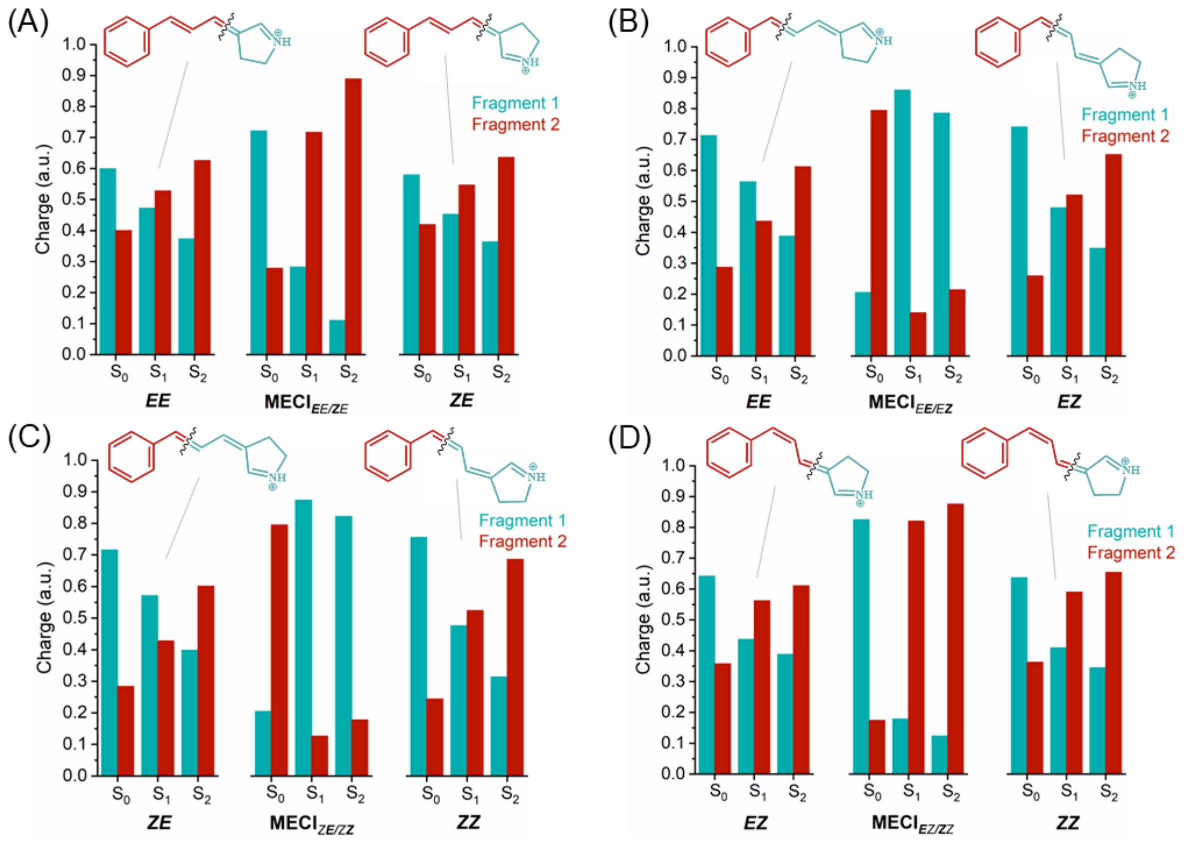

Figure S3. Charge distribution on the molecular fragments of ExBP at each side of the isomerizing double bond for various isomers and MECI points in S_0, S_1, and S_2 for **(A)** ***EE***→***ZE***, **(B)** ***EE***→***EZ***, **(C)** ***ZE***→***ZZ***, and **(D)** ***EZ***→***ZZ*** isomerizations.

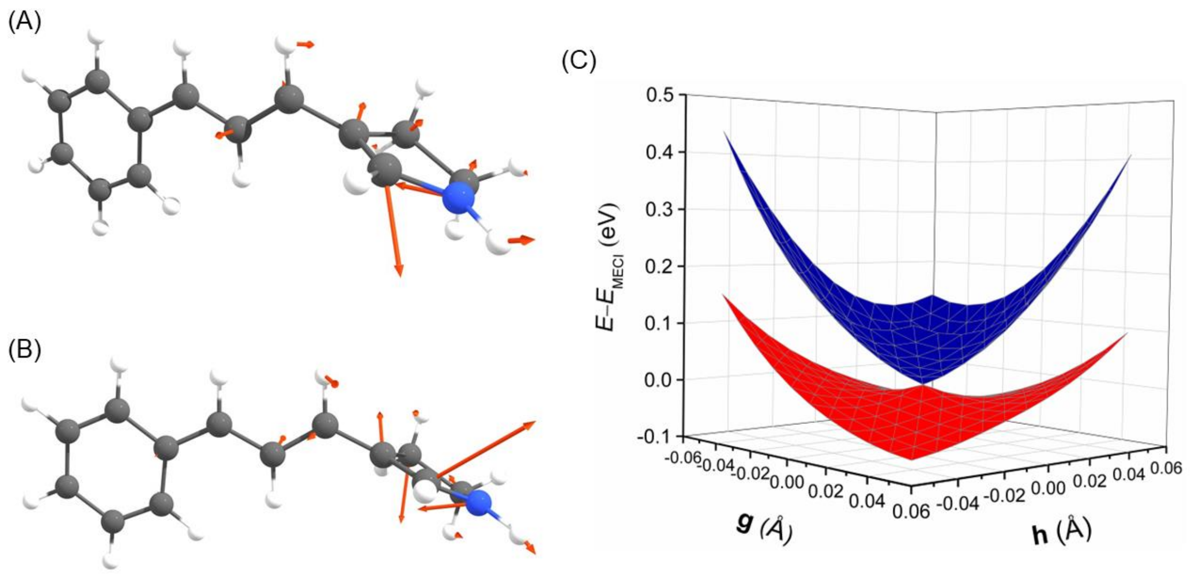

Figure S4. ***EE***→***ZE*** isomerization of ExBP. **(A)** Gradient difference (GDV; **g**) and **(B)** nonadiabatic coupling (NACV; **h**) orthonormalized vectors at the minimum energy conical intersection. **(C)** 2D potential energy surfaces (in eV) of S_0 and S_1 along two branching vectors centered around the minimum energy conical intersection.

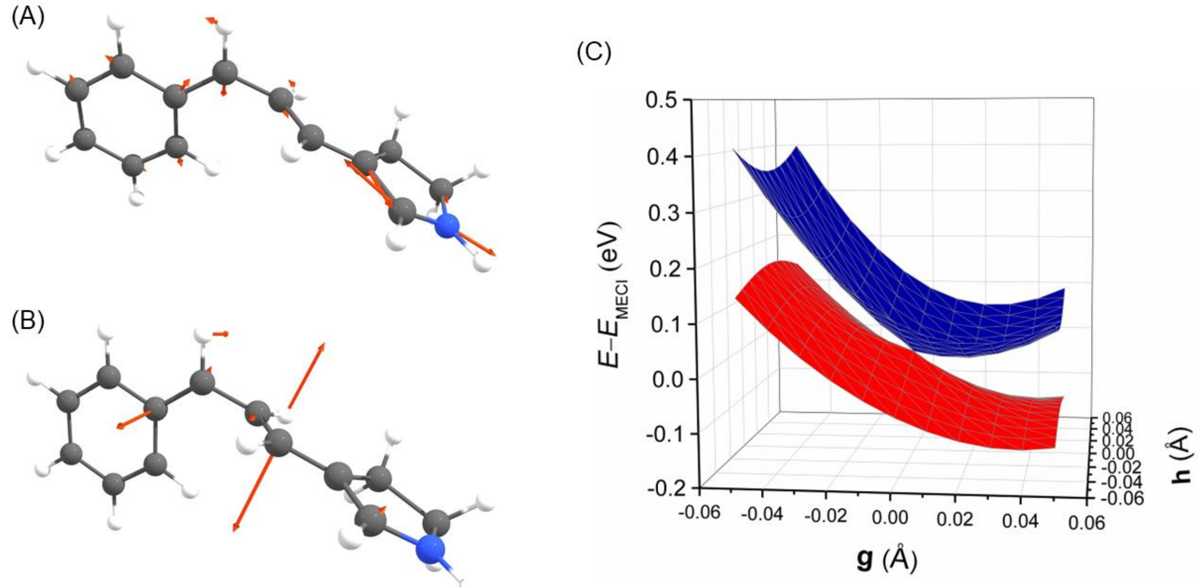

Figure S5. ***EE***$\rightarrow$***EZ*** isomerization of ExBP. **(A)** Gradient difference (GDV; **g**) and **(B)** nonadiabatic coupling (NACV; **h**) orthonormalized vectors at the minimum energy conical intersection. **(C)** 2D potential energy surfaces (in eV) of S_0 and S_1 along two branching vectors centered around the minimum energy conical intersection.

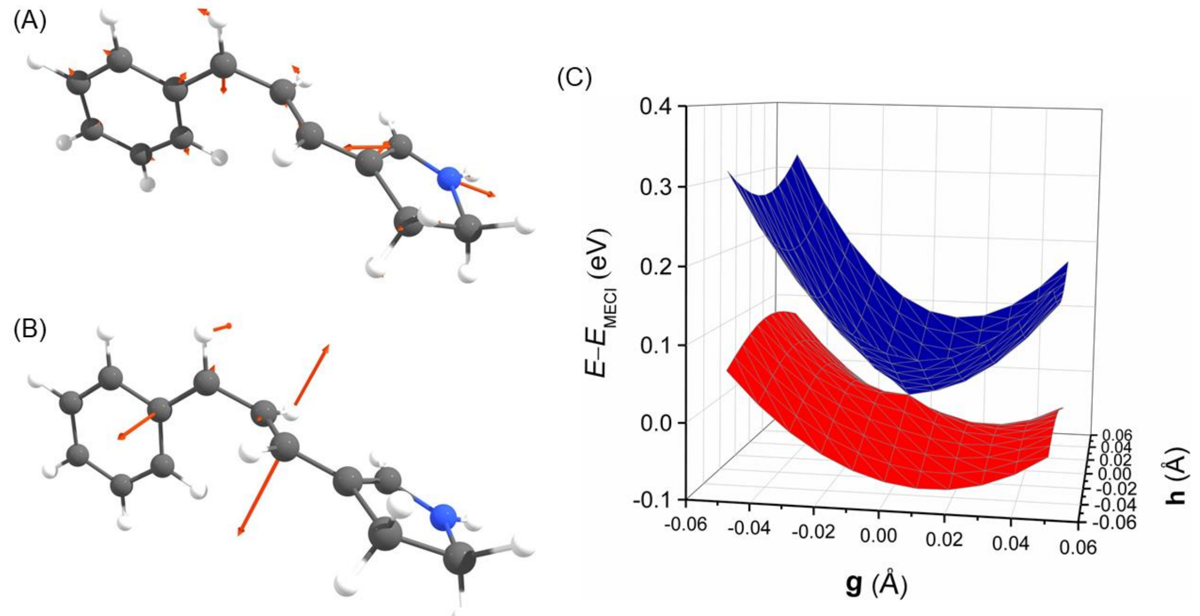

Figure S6. ***ZE***$\rightarrow$***ZZ*** isomerization of ExBP. **(A)** Gradient difference (GDV; **g**) and **(B)** nonadiabatic coupling (NACV; **h**) orthonormalized vectors at the minimum energy conical intersection. **(C)** 2D potential energy surfaces (in eV) of S_0 and S_1 along two branching vectors centered around the minimum energy conical intersection.

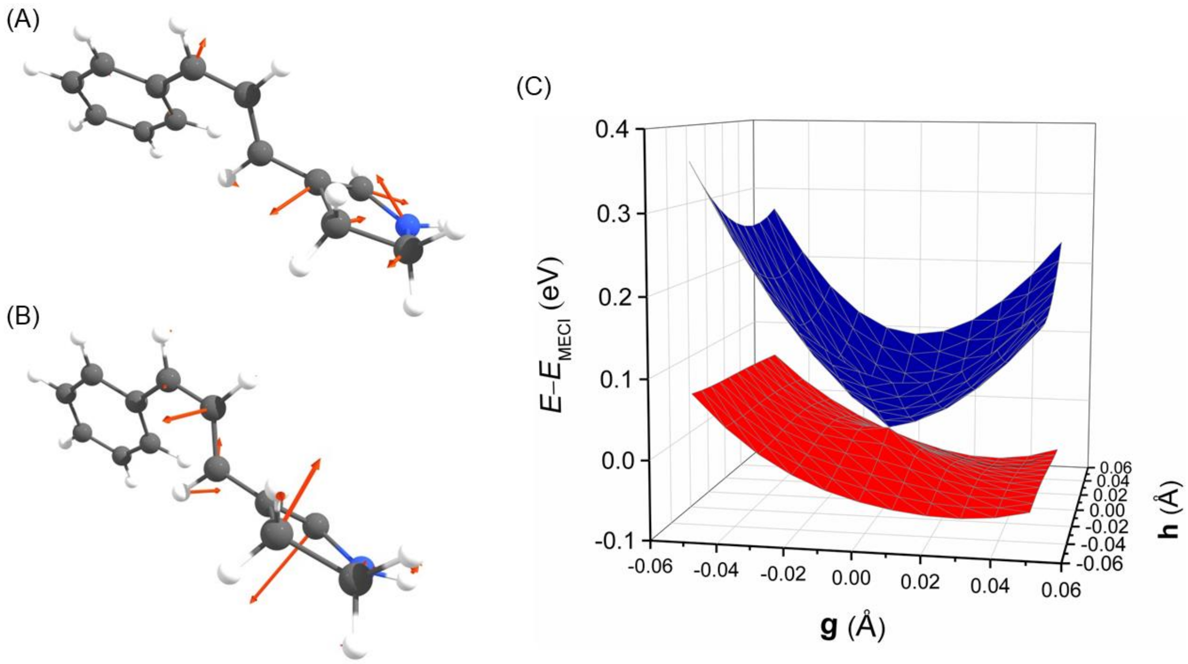

Figure S7. ***E**Z*→***Z**Z* isomerization of ExBP. **(A)** Gradient difference (GDV; **g**) and **(B)** nonadiabatic coupling (NACV; **h**) orthonormalized vectors at the minimum energy conical intersection. **(C)** 2D potential energy surfaces (in eV) of S_0 and S_1 along two branching vectors centered around the minimum energy conical intersection.

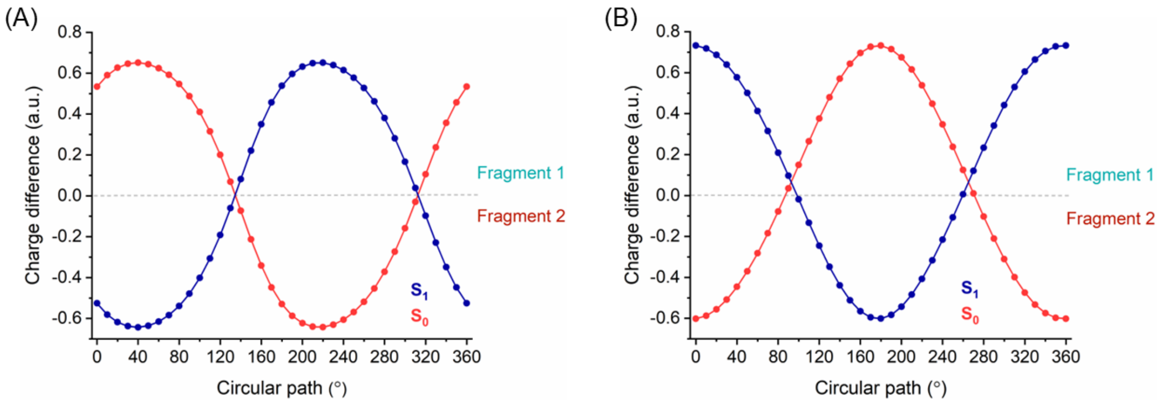

Figure S8. Difference in charge distribution between molecular fragments (i.e., “fragment 1 charge” minus “fragment 2 charge”) for a varying circular loop angle around the minimum energy conical intersection for **(A)** ***E**E*→***Z**E* and **(B)** *E**E***→*E**Z*** isomerizations.

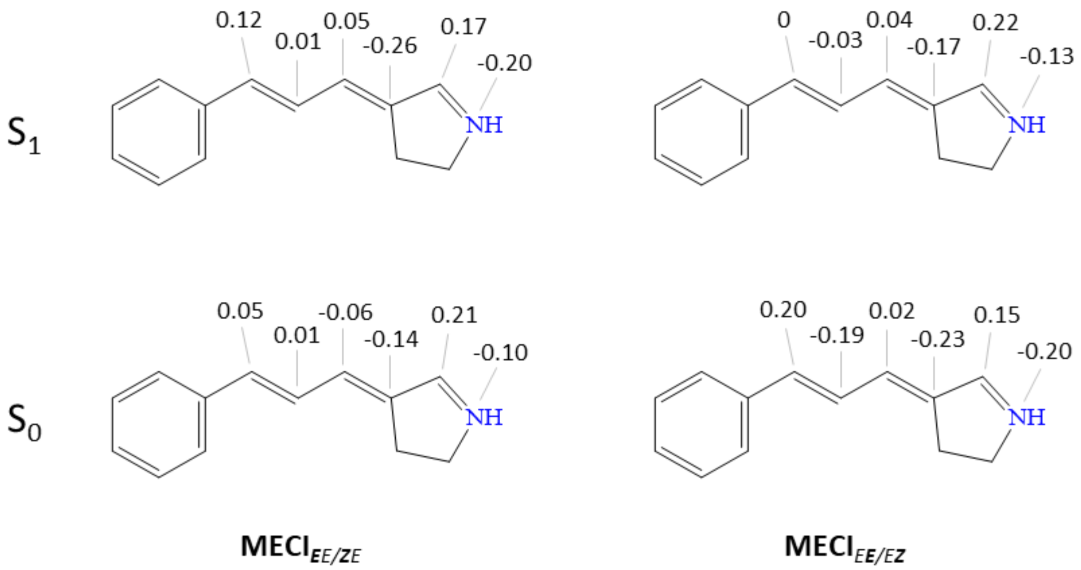

Figure S9. Ground (S_0) and first excited state (S_1) atomic charges (a.u.) at minimum energy conical intersections for ***EE*↔*ZE***, and ***EE*↔*EZ*** isomerizations.

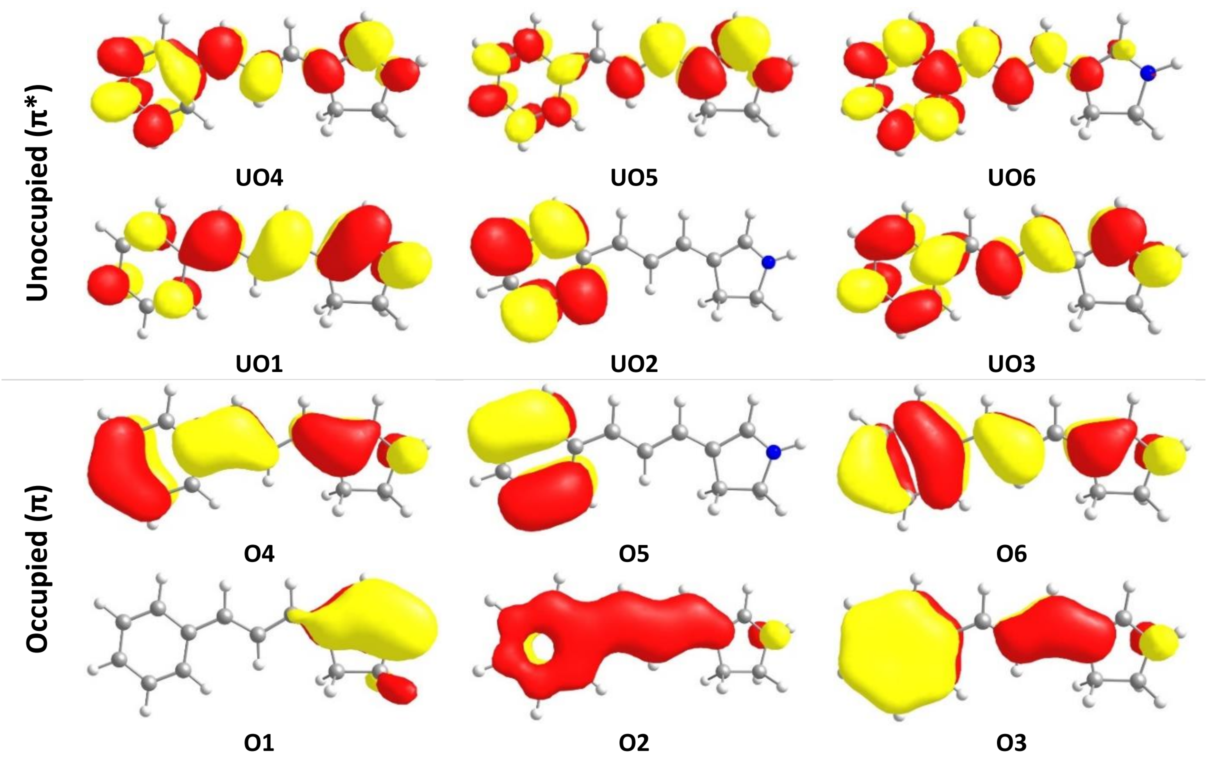

Figure S10. Active space orbitals (12,12) of ExBP used in the XMS-CASPT2 calculations. "O" and "UO" refer to the occupied and unoccupied orbitals, while the positive and negative lobes of the orbitals are shown in yellow and red, respectively.

Table S1. Vertical excitation energies (wavelengths) and oscillator strengths (*f*) for *E* and *Z* isomers of 4-benzylidene-3,4-dihydro-2H-pyrrolium (BP) at various levels of theories using cc-pVDZ and cc-pVTZ basis sets

Method	Excitation	*E* isomer[a]				*Z* isomer			
		cc-pVDZ		cc-pVTZ		cc-pVDZ		cc-pVTZ	
		ΔE_{calc} eV (nm)	*f*	ΔE_{calc} eV (nm)	*f*	ΔE_{calc} eV (nm)	*f*	ΔE_{calc} eV (nm)	*f*
XMS-CASPT2	$S_0 \rightarrow S_1$	3.46 (358)	1.02	3.35 (370)	1.03	3.42 (363)	0.35	3.29 (377)	0.35
	$S_0 \rightarrow S_2$	3.66 (339)	0.01	3.54 (350)	0.01	3.66 (339)	0.00	3.52 (352)	0.01
	$S_0 \rightarrow S_3$	4.87 (255)	0.08	4.75 (261)	0.07	4.94 (251)	0.01	4.61 (269)	0.01
CC2	$S_0 \rightarrow S_1$	3.59 (345)	0.89	3.51 (353)	0.88	3.47 (357)	0.34	3.37 (368)	0.33
	$S_0 \rightarrow S_2$	3.77 (328)	0.01	3.69 (336)	0.01	3.77 (329)	0.01	3.66 (339)	0.01
	$S_0 \rightarrow S_3$	5.48 (226)	0.07	5.36 (231)	0.06	5.11 (242)	0.19	4.98 (249)	0.18
ADC(2)	$S_0 \rightarrow S_1$	3.45 (359)	0.84	3.37 (368)	0.82	3.40 (364)	0.33	3.30 (376)	0.31
	$S_0 \rightarrow S_2$	3.75 (331)	0.02	3.66 (339)	0.02	3.75 (331)	0.01	3.64 (341)	0.01
	$S_0 \rightarrow S_3$	5.39 (230)	0.06	5.27 (235)	0.06	5.01 (248)	0.19	4.87 (255)	0.18

[a]The experimental absorption (ΔE_{exp}) for the *E* isomer is found to be 3.80 eV (326 nm) and 5.52 eV (225 nm) with approximate oscillator strengths of 0.7 and 0.1 for $S_0 \rightarrow S_1$ and $S_0 \rightarrow S_3$ transitions, respectively in solution.[1]

Note: While all the methods considered in this study exhibit red-shifted excitation energies compared to the experimentally observed values, notably, the XMS-CASPT2 and ADC(2) methods yield similar results. The excitation energies obtained with the cc-pVTZ basis set are slightly more red-shifted compared to the cc-pVDZ values.

Table S2. Emission energies (wavelengths) and oscillator strengths (*f*) for *E* and *Z* isomers of 4-benzylidene-3,4-dihydro-2H-pyrrolium (BP) at various levels of theories using cc-pVDZ and cc-pVTZ basis sets

Method	Emission	*E* isomer				*Z* isomer			
		cc-pVDZ		cc-pVTZ		cc-pVDZ		cc-pVTZ	
		ΔE_{calc} eV (nm)	*f*	ΔE_{calc} eV (nm)	*f*	ΔE_{calc} eV (nm)	*f*	ΔE_{calc} eV (nm)	*f*
XMS-CASPT2	$S_1 \rightarrow S_0$	3.19 (388)	1.07	3.09 (402)	1.07	2.80 (442)	0.65	2.69 (461)	0.64
CC2	$S_1 \rightarrow S_0$	3.35 (370)	0.94	3.28 (378)	0.92	2.94 (422)	0.57	2.85 (435)	0.54
ADC(2)	$S_1 \rightarrow S_0$	3.14 (394)	0.89	3.07 (403)	0.87	2.75 (452)	0.55	2.66 (466)	0.52

Note: In the case of the *E* isomer, the emission maximum is red-shifted by 25-35 nm compared to the absorption maximum (**Table S1**) for various methods considered here. The red-shift is more significant for the *Z* isomer, ranging in 65-90 nm for two basis sets considered here.

Table S3. Vertical excitation energies and oscillator strengths (*f*) for different possible isomers of ExBP at various levels of theories using cc-pVDZ basis set

Method	Excitation	*EE*		*ZE*		*EZ*		*ZZ*	
		ΔE_{calc} eV (nm)	*f*	ΔE_{calc} eV (nm)	*f*	ΔE_{calc} eV (nm)	*f*	ΔE_{calc} eV (nm)	*f*
XMS-CASPT2	$S_0 \rightarrow S_1$	2.93 (423)	1.58	2.83 (438)	1.24	3.19 (389)	0.82	3.15 (393)	0.74
	$S_0 \rightarrow S_2$	3.58 (346)	0.01	3.58 (347)	0.01	3.72 (333)	0.00	3.78 (328)	0.00
	$S_0 \rightarrow S_3$	4.00 (310)	0.05	3.99 (311)	0.12	4.13 (300)	0.29	4.10 (303)	0.19
CC2	$S_0 \rightarrow S_1$	3.02 (410)	1.39	2.92 (425)	1.12	3.15 (393)	0.65	3.10 (401)	0.57
	$S_0 \rightarrow S_2$	3.68 (337)	0.02	3.69 (336)	0.02	3.70 (335)	0.00	3.68 (337)	0.00
	$S_0 \rightarrow S_3$	4.74 (262)	0.09	4.69 (264)	0.09	4.35 (285)	0.35	4.18 (297)	0.24
ADC(2)	$S_0 \rightarrow S_1$	2.86 (433)	1.24	2.77 (447)	0.99	3.06 (405)	0.63	3.02 (411)	0.55
	$S_0 \rightarrow S_2$	3.67 (338)	0.02	3.69 (336)	0.02	3.70 (335)	0.00	3.68 (337)	0.00
	$S_0 \rightarrow S_3$	4.61 (269)	0.07	4.58 (270)	0.08	4.23 (293)	0.30	4.09 (303)	0.21

Table S4. Vertical excitation energies and oscillator strengths (*f*) for different possible isomers of ExBP at various levels of theories using cc-pVTZ basis set

Method	Excitation	*EE*		*ZE*		*EZ*		*ZZ*	
		ΔE_{calc} eV (nm)	*f*	ΔE_{calc} eV (nm)	*f*	ΔE_{calc} eV (nm)	*f*	ΔE_{calc} eV (nm)	*f*
XMS-CASPT2	$S_0 \rightarrow S_1$	2.84 (436)	1.60	2.73 (455)	1.24	3.10 (401)	0.83	3.05 (407)	0.74
	$S_0 \rightarrow S_2$	3.46 (359)	0.01	3.45 (359)	0.01	3.62 (343)	0.00	3.67 (338)	0.00
	$S_0 \rightarrow S_3$	3.89 (318)	0.05	3.87 (321)	0.11	4.00 (310)	0.28	3.96 (313)	0.18
CC2	$S_0 \rightarrow S_1$	2.96 (419)	1.37	2.84 (436)	1.09	3.07 (403)	0.64	3.00 (413)	0.55
	$S_0 \rightarrow S_2$	3.59 (345)	0.02	3.60 (344)	0.02	3.60 (344)	0.00	3.58 (346)	0.01
	$S_0 \rightarrow S_3$	4.63 (268)	0.09	4.58 (271)	0.08	4.24 (292)	0.34	4.06 (305)	0.22
ADC(2)	$S_0 \rightarrow S_1$	2.79 (444)	1.21	2.70 (460)	0.95	2.98 (416)	0.60	2.93 (424)	0.52
	$S_0 \rightarrow S_2$	3.57 (347)	0.02	3.60 (345)	0.02	3.59 (345)	0.00	3.58 (347)	0.00
	$S_0 \rightarrow S_3$	4.50 (275)	0.06	4.47 (278)	0.08	4.12 (301)	0.29	3.97 (312)	0.20

Table S5. Topology parameters for MECIs of *EE*→*ZE* and *EE*→*EZ* isomerizations. The values are in atomic units and radians.

MECI	$\boldsymbol{\delta_{gh}}$	$\boldsymbol{\Delta_{gh}}$	$\boldsymbol{\sigma}$	$\boldsymbol{\theta_s}$	P	B	type
EE*→*ZE	0.0476	−0.5512	0.0152	0.6324	0.00	0.09	peaked bifurcating
EE*→*EZ	0.0546	−0.6137	1.4226	0.6584	3.75	1.62	sloped single-path

Note: Peaked intersection (P < 1); sloped intersection (P > 1); single-path (B > 1); bifurcating (B < 1). Pitch $\boldsymbol{\delta_{gh}}$, asymmetry $\boldsymbol{\Delta_{gh}}$, relative tilt $\boldsymbol{\sigma}$, tilt heading $\boldsymbol{\theta_s}$ as described in reference S2.

Reference

(S1) Sampedro, D.; Migani, A.; Pepi, A.; Busi, E.; Basosi, R.; Latterini, L.; Elisei, F.; Fusi, S.; Ponticelli, F.; Zanirato, V.; Olivucci, M. Design and Photochemical Characterization of a Biomimetic Light-Driven Z/E Switcher. *J. Am. Chem. Soc.* **2004**, *126* (30), 9349–9359. https://doi.org/10.1021/ja038859e.

(S2) Fdez. Galván, I.; Delcey, M. G.; Pedersen, T. B.; Aquilante, F.; Lindh, R. Analytical State-Average Complete-Active-Space Self-Consistent Field Nonadiabatic Coupling Vectors: Implementation with Density-Fitted Two-Electron Integrals and Application to Conical Intersections. *J. Chem. Theory Comput.* **2016**, *12* (8), 3636–3653. https://doi.org/10.1021/acs.jctc.6b00384.

Geometry optimized coordinates of BP and ExBP at the XMS-CASPT2/cc-pVDZ level of theory.

BP

E

C	-2.200480240117	1.314956268507	-0.000416507466
C	-1.105545745493	0.412484965734	0.000421368108
C	-1.367317037550	-0.982880506993	-0.018862715213
C	-2.687112223912	-1.450459502718	-0.038308063124
C	-3.762942483810	-0.541874699052	-0.039343139842
C	-3.519181918114	0.842618839018	-0.020373365918
H	-2.006872181026	2.393008505414	0.014667014849
H	-0.551396217716	-1.706708083861	-0.017926954132
H	-2.880677660734	-2.526658708754	-0.052656876022
H	-4.790913223065	-0.915618163722	-0.054732816056
H	-4.353247630130	1.549591430572	-0.021010172859
C	0.228767425755	0.992822181351	0.020892551584
H	0.251578216472	2.090404152823	0.039097037742
C	2.661845894860	1.147678050989	0.039237104025
H	2.746346167957	2.238076040519	0.057917886223
H	4.689089319072	0.747313699994	0.042423098233
C	1.461979135933	0.375212294493	0.019722530257
N	3.736330015949	0.381497163075	0.031695557021
C	1.872010822175	-1.091788931936	-0.003393918994
H	1.492359431545	-1.596013921203	-0.905494327327
H	1.482983800086	-1.627374720768	0.876310076505
C	3.423124899454	-1.063248752939	0.006377533142
H	3.873430786070	-1.510689586025	-0.892475871466
H	3.862118004740	-1.538943725242	0.896298445810

Z

C	-2.107678171689	-1.256525987451	-0.271505971853
C	-1.079887428368	-0.461325347914	0.278588750162
C	-1.322732541598	0.900079531539	0.562133007289
C	-2.573088551248	1.469644870276	0.263186608014
C	-3.579635892756	0.682698800110	-0.317904395934
C	-3.347376904080	-0.679503378577	-0.583874819062
H	-1.929692651039	-2.319453242880	-0.469284938416
H	-0.553539564822	1.497073741986	1.067331900283
H	-2.763702914964	2.521144255359	0.499109859182
H	-4.553872166324	1.124604985912	-0.547570157808
H	-4.138166129446	-1.294740488949	-1.023570637892
C	0.236529292051	-1.074055598940	0.578241058421
H	0.242562690015	-2.032609208921	1.113784761932
C	1.576884773125	0.658812139253	-0.588630562324
H	0.785688360266	1.209020129309	-1.106010976455
H	3.164874333233	1.793028962068	-1.260674367604
C	1.436487698882	-0.517294687422	0.232481314437
N	2.845918043416	0.995241655580	-0.707336221482

C	2.844715030159	-1.020980647710	0.529374094470
H	3.059834135881	-1.917366106964	-0.074812748305
H	2.981593298389	-1.278112343370	1.589027684502
C	3.757173446359	0.152813391821	0.103816509235
H	4.619178730268	-0.147772440717	-0.508007724474
H	4.115467069609	0.752320458198	0.956594438302

MECI$_{E/Z}$

C	-3.496581716400	0.386585428839	2.760060153623
C	-2.444331068144	0.194797641318	1.800113469404
C	-2.759016664152	0.151066930427	0.398063162018
C	-4.077896577940	0.304943979774	-0.018272076554
C	-5.099479745238	0.500305240750	0.943920617566
C	-4.811992738182	0.540262409645	2.328635139794
H	-3.246331265729	0.414348903401	3.825881798863
H	-1.950050097164	-0.014587485705	-0.320984372071
H	-4.331826415142	0.272524681713	-1.081024819590
H	-6.134341571267	0.620387314528	0.608108452477
H	-5.619270225166	0.689496784073	3.050141885708
C	-1.117242556111	0.046712849829	2.250279784232
H	-0.957488172845	0.077273066103	3.340805521488
C	0.626587570346	-1.180803103207	0.846553854930
H	0.337977938897	-2.222553272767	1.009689692585
H	2.346529826058	-1.567570257726	-0.259318415977
C	0.044848207320	-0.051871420527	1.373331757149
N	1.645343166531	-0.870910758652	-0.012047391037
C	0.739242038204	1.171022740766	0.762612519226
H	0.973947121198	1.953984973304	1.500891876144
H	0.092267438322	1.622921949899	-0.013505545842
C	2.013474428390	0.545590944575	0.149670395992
H	2.866729671080	0.631792513966	0.847547225609
H	2.292669517485	0.992613686642	-0.815654826413

ExBP

EE

C	-3.410784649715	-1.231655347270	-0.000226352573
C	-2.308192615460	-0.343578173154	0.000051307281
C	-2.552001771590	1.053598030086	0.000353922678
C	-3.863837534993	1.540364719630	0.000351291516
C	-4.952407796307	0.645102383840	0.000053668796
C	-4.725032495443	-0.741448823227	-0.000236230736
H	-3.229735333325	-2.312184966806	-0.000448242857
H	-1.717975726453	1.761784997030	0.000609148954
H	-4.043963797616	2.619191992957	0.000592883739
H	-5.975746348576	1.031714702427	0.000054733072
H	-5.568313366212	-1.437753657347	-0.000460379538

C	3.943142511963	-1.041565861872	0.000038525346
H	4.079499923659	-2.126753089829	0.000138802273
H	5.953778735658	-0.544393262114	-0.000109067878
C	2.719985971014	-0.325859518609	0.000004859770
N	4.985562886374	-0.224002130195	-0.000054760840
C	3.052393888707	1.160753662400	0.000105628907
H	2.640935621293	1.661259726101	0.890702973892
H	2.640308794522	1.661613204143	-0.889995560772
C	4.603790203414	1.204995813107	-0.000429790289
H	5.025883090067	1.688603282681	0.893581645251
H	5.025253575376	1.687892649536	-0.895131535678
C	1.475690030645	-0.933951764180	0.000015642089
H	1.441086466544	-2.031182853211	0.000000398361
C	0.231423351210	-0.230340163159	0.000032006285
H	0.251643645283	0.864116158826	0.000049672079
C	-0.965079113786	-0.913858222876	0.000025683592
H	-0.916144362792	-2.011234381331	-0.000030120988

ZE

C	-3.409010483140	-1.235480369696	0.015900710209
C	-2.320946676578	-0.331971139112	0.000231690798
C	-2.586913721396	1.059788126265	-0.008918835583
C	-3.906047327188	1.527122941540	-0.001754013187
C	-4.980702847903	0.616308905806	0.014458251686
C	-4.731109037745	-0.765595955665	0.023193977429
H	-3.212127078708	-2.313212400043	0.022623983833
H	-1.764631223347	1.781763339529	-0.022506815207
H	-4.102098524580	2.603240508199	-0.009104791880
H	-6.009822053662	0.987134682608	0.019906357390
H	-5.562992194271	-1.475468177414	0.035516936170
C	3.036786129246	1.067191697908	-0.043437532304
H	2.342377324949	1.910112602134	-0.082260788259
H	4.761596255572	2.211367668491	0.005854345997
C	2.736724314754	-0.321096850383	-0.027264622876
N	4.344830397937	1.280464403713	-0.006453535841
C	4.064891557724	-1.072026353487	-0.055738857610
H	4.189127697133	-1.594491798246	-1.018011994970
H	4.132066327974	-1.820326004377	0.747807406716
C	5.134631389596	0.035369440308	0.111940934844
H	5.912836636568	0.020274135265	-0.664770206886
H	5.619945343058	0.021773053570	1.101068021528
C	1.469350736898	-0.884077221277	-0.020665364632
H	1.416234150152	-1.980660880422	-0.016247212251
C	0.218138607350	-0.185508983204	-0.012303893711
H	0.214614543949	0.909265477260	-0.010416045012
C	-0.967026217116	-0.883973543353	-0.005717265979
H	-0.903541537346	-1.980336206434	-0.003839520784

EZ

C	3.241486495504	-0.944440173062	0.502324100448
C	2.111685503755	-0.674076281127	-0.301682802051
C	2.018544234225	0.564482251940	-0.975654783456
C	3.027652818274	1.530428313690	-0.821996185616
C	4.131909547849	1.267039399380	0.006494973507
C	4.237797122155	0.028831637730	0.667876851218
H	3.328914439259	-1.912596675714	1.008391244173
H	1.178634322032	0.748778373741	-1.656046667988
H	2.958664962523	2.481688271385	-1.359550787078
H	4.918793460653	2.018416405033	0.124537634170
H	5.104440874557	-0.181246951059	1.302496264653
C	-2.734706161961	1.226584170734	0.704712711255
H	-2.180643041043	2.089123163358	1.087402720455
H	-4.630380089060	2.012049736353	0.971533471562
C	-2.198623348943	0.006439025267	0.206077558765
N	-4.056156483923	1.226907712806	0.661671743454
C	-3.369488382985	-0.886482429676	-0.185533069368
H	-3.381096419669	-1.807469445203	0.418489394911
H	-3.316022731159	-1.177943431404	-1.245604477072
C	-4.624114792655	-0.018767180570	0.097345343231
H	-5.309614274769	-0.465376883318	0.833136599352
H	-5.194072450734	0.231674636847	-0.810714899874
C	-0.845582761819	-0.245002265762	0.127710935477
H	-0.151375430292	0.536010815610	0.461676853429
C	-0.285041315768	-1.496977689511	-0.324243999487
H	-0.972322571156	-2.327825925643	-0.517446224751
C	1.061750689273	-1.709667827820	-0.460595199018
H	1.399550454898	-2.723985786913	-0.707032939542

ZZ

C	3.204119719228	-0.911749282225	0.528157877712
C	2.100466627584	-0.697648856376	-0.327807001264
C	2.022793921182	0.497745299308	-1.077624840451
C	3.022765854514	1.477128458456	-0.951963718804
C	4.102865661899	1.269660646115	-0.076696391632
C	4.192299429673	0.074163048085	0.662535518120
H	3.277234382044	-1.845812941680	1.096794508880
H	1.199799885873	0.637918517666	-1.788623059637
H	2.964694845539	2.395743994321	-1.544760035554
H	4.882709163348	2.031433412225	0.019871496736
H	5.039124271902	-0.091478048766	1.335789961233
C	-3.262594603187	-0.808021245422	-0.145742804694
H	-3.222282520047	-1.805907034334	-0.590131883510
H	-5.323879118032	-0.691841926680	-0.059051191157
C	-2.175670154081	0.044701890786	0.217150563366
N	-4.425224509403	-0.239049365295	0.116799275175
C	-2.777911850659	1.363639677996	0.693449467251

H	-2.566722941814	2.163215680569	-0.034715843191
H	-2.372513745991	1.673923037247	1.667479237227
C	-4.298021284627	1.083148430207	0.774599147628
H	-4.916379042100	1.817042924166	0.238190264467
H	-4.668558977240	0.991350405490	1.808238043447
C	-0.838022912049	-0.267057013880	0.108383489899
H	-0.115591847716	0.505685940172	0.401696556025
C	-0.288117798542	-1.539180510695	-0.312293799795
H	-0.965901924172	-2.384770270470	-0.471501664097
C	1.056145281232	-1.746732758685	-0.459133350998
H	1.398124781220	-2.761942834889	-0.694952255129

MECI*$_{EE/ZE}$*

C	-2.0294082420	-4.1273981823	1.3303958967
C	-0.6831062010	-3.7026146243	1.5399124506
C	0.1363053164	-4.4313964369	2.4549986940
C	-0.3804838310	-5.5396030770	3.1288090003
C	-1.7159109893	-5.9457200605	2.9085131962
C	-2.5391898746	-5.2383878580	2.0087928868
H	-2.6607943249	-3.5708061711	0.6291081001
H	1.1714959042	-4.1264008932	2.6339989559
H	0.2493900042	-6.0954921276	3.8291891609
H	-2.1126970654	-6.8153933231	3.4407939973
H	-3.5718073481	-5.5573025370	1.8423992662
C	3.1408217675	0.8279118327	0.8905984172
H	2.5792995791	1.3698022068	1.6564014079
H	4.9450055553	1.8718141752	1.1160011283
C	2.7187942272	-0.2416083022	0.1236875048
N	4.4097829503	1.1949625352	0.5742036155
C	3.8217938961	-0.5751924897	-0.8758872071
H	3.5166935869	-0.2520982901	-1.8888117599
H	4.0359988714	-1.6549099673	-0.9254000253
C	5.0294101347	0.2416092877	-0.3583929002
H	5.5721023575	0.7759968868	-1.1523960833
H	5.7451941372	-0.3947869938	0.1928957791
C	1.3894089439	-0.8585964833	0.1221989344
H	0.6144998112	-0.4385923196	-0.5395094645
C	1.0738970154	-1.9816111355	0.8779107269
H	1.8431979543	-2.4080997539	1.5300971905
C	-0.2144946468	-2.5584819146	0.8152894275
H	-0.9356995131	-2.0771039791	0.1405017421

MECI*$_{EE/EZ}$*

C	-3.3278241882	-1.2506349857	-1.1105683141
C	-2.0842767134	-0.5422337496	-1.0924494429
C	-1.8805374225	0.4560514786	-2.0930778239
C	-2.8611358583	0.7258645655	-3.0548689350
C	-4.0772371154	0.0131241675	-3.0607537043

C	-4.2993864457	-0.9750957398	-2.0795022868
H	-3.5209377257	-2.0231580636	-0.3578558400
H	-0.9421613953	1.0231226809	-2.1115701080
H	-2.6778988748	1.4969122250	-3.8088666665
H	-4.8397258669	0.2263597283	-3.8129279876
H	-5.2416416132	-1.5326589427	-2.0726754415
C	3.8029796637	-0.2048892955	-1.1334653093
H	3.9586339436	-1.0487505964	-1.8118288389
H	5.7235240214	0.5645675546	-1.2375228974
C	2.6042417082	0.1616633274	-0.4583335247
N	4.7797642227	0.6449169723	-0.8507984971
C	2.8946341080	1.4076691150	0.3558190099
H	2.7175136128	1.2183716170	1.4265240303
H	2.2398812465	2.2346271304	0.0412436569
C	4.3811930846	1.7236181528	0.0741749665
H	5.0226091400	1.6743912658	0.9682899255
H	4.5418573118	2.6922111861	-0.4255802731
C	1.3840970473	-0.5510330258	-0.5977268306
H	1.3796942590	-1.4485106259	-1.2278625816
C	0.2039615423	-0.1482784266	0.0122272256
H	0.2629645041	0.7534738670	0.6462551464
C	-1.0811405037	-0.8711810166	-0.1219827686
H	-1.2994458616	-1.6005205714	0.6649840948

MECI$_{ZE/ZZ}$

C	-3.3563149414	-1.4997468461	-0.6370673297
C	-2.1554595441	-0.7399435302	-0.8196151331
C	-2.1212792865	0.2376367973	-1.8665316254
C	-3.2376476582	0.4442104147	-2.6797890478
C	-4.4127674575	-0.3150096347	-2.4835744733
C	-4.4641268487	-1.2863268602	-1.4595784703
H	-3.3938112653	-2.2501232211	0.1593173219
H	-1.2107748396	0.8256000037	-2.0252850646
H	-3.2014956601	1.1956098190	-3.4737076355
H	-5.2829781791	-0.1498080821	-3.1252776445
H	-5.3744165062	-1.8731120567	-1.3088946566
C	2.8866128266	1.3048617194	-0.2713228195
H	2.2919098722	1.9141629916	0.4129780623
H	4.5825576738	2.5091665160	-0.2246624930
C	2.5288875321	0.1068385991	-0.9193625731
N	4.1321762616	1.6706606799	-0.5922465095
C	3.7044878179	-0.3312891382	-1.7729298824
H	3.4152221229	-0.3675967442	-2.8368161863
H	4.0389298494	-1.3427256938	-1.4892926397
C	4.7966857056	0.7322989512	-1.5112158310
H	5.1099170397	1.2713552074	-2.4189320091
H	5.6939213003	0.3213736433	-1.0214328823
C	1.2921564952	-0.5977821249	-0.8234090630

H	1.2025991788	-1.5202008638	-1.4094970012
C	0.2154085040	-0.2034717713	-0.0546551634
H	0.2877507759	0.7220436590	0.5393506568
C	-1.0374721681	-0.9749959915	0.0253902652
H	-1.1221785999	-1.7233863896	0.8256598306

MECI$_{EZ/ZZ}$

C	3.4410120935	-0.5912991260	-0.5857959160
C	2.1199030018	-0.9014286332	-0.1389685036
C	1.5622102426	-2.1840013401	-0.4314823641
C	2.2855173117	-3.0939032981	-1.2059499590
C	3.5667880819	-2.7472702955	-1.6902126606
C	4.1453739078	-1.4981208204	-1.3797342401
H	3.8789455906	0.3789659991	-0.3277915038
H	0.6093163694	-2.4782211357	0.0170423616
H	1.8746686846	-4.0852809316	-1.4164670453
H	4.1310077013	-3.4691580628	-2.2889831597
H	5.1461031057	-1.2512002604	-1.7441678508
C	-3.2666018071	-0.9544236144	0.9362428301
H	-3.0493214224	-1.9069360880	1.4293273901
H	-5.2865362443	-0.8535340372	1.4647422040
C	-2.3782242162	-0.1031219472	0.3261750599
N	-4.5561173016	-0.5127004460	0.8426844015
C	-3.1576842918	1.0772730329	-0.2561053868
H	-3.1191733626	1.0755100569	-1.3601999163
H	-2.7579922523	2.0475552388	0.0860804931
C	-4.6036216293	0.8387436414	0.2599110158
H	-5.3433118310	0.8701550264	-0.5535409310
H	-4.8908242614	1.5868635399	1.0195377844
C	-0.9535876560	-0.3395238701	0.0673046856
H	-0.6514599871	-0.8857659868	-0.8400188467
C	0.0386998756	0.2258253311	0.8569454064
H	-0.2740517918	0.9073356757	1.6569771026
C	1.4299057173	0.1029976059	0.6125447244
H	2.0680555589	0.8763699673	1.0614017694
